# Deep Learning-Based Morphological Classification of Human Sperm Heads

**DOI:** 10.3390/diagnostics10050325

**Published:** 2020-05-20

**Authors:** Imran Iqbal, Ghulam Mustafa, Jinwen Ma

**Affiliations:** 1 Department of Information and Computational Sciences, School of Mathematical Sciences and LMAM, Peking University, Beijing 100871, China; imraniqbalrajput@pku.edu.cn; 2 Department of Biomedical Engineering, College of Engineering, Peking University, Beijing 100871, China; mustafabme@gmail.com

**Keywords:** classification, convolutional neural network (CNN), deep learning, infertility, sperm head morphology

## Abstract

Human infertility is considered as a serious disease of the reproductive system that affects more than 10% of couples across the globe and over 30% of the reported cases are related to men. The crucial step in the assessment of male infertility and subfertility is semen analysis that strongly depends on the sperm head morphology, i.e., the shape and size of the head of a spermatozoon. However, in medical diagnosis, the morphology of the sperm head is determined manually, and heavily depends on the expertise of the clinician. Moreover, this assessment as well as the morphological classification of human sperm heads are laborious and non-repeatable, and there is also a high degree of inter and intra-laboratory variability in the results. In order to overcome these problems, we propose a specialized convolutional neural network (CNN) architecture to accurately classify human sperm heads based on sperm images. It is carefully designed with several layers, and multiple filter sizes, but fewer filters and parameters to improve efficiency and effectiveness. It is demonstrated that our proposed architecture outperforms state-of-the-art methods, exhibiting 88% recall on the SCIAN dataset in the total agreement setting and 95% recall on the HuSHeM dataset for the classification of human sperm heads. Our proposed method shows the potential of deep learning to surpass embryologists in terms of reliability, throughput, and accuracy.

## 1. Introduction

**Figure 1:**
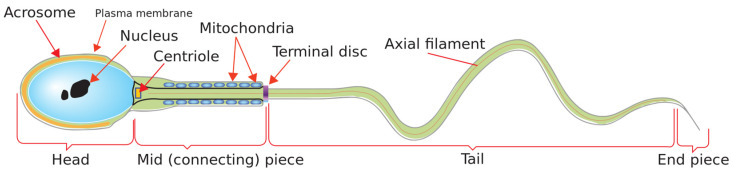
The diagram of a human spermatozoon.

Human spermatozoon is the gamete−the male reproductive cell−that may fertilize the mature oocyte. It is produced in the seminiferous tubules of the testicles. Structurally, normal human spermatozoa have four main parts: head, midpiece, tail, and end piece, as shown in Figure 1 [1]. A normal human sperm has a smooth oval head which looks like the shape of an egg. The sperm head can be further divided into two subunits: nucleus and acrosome.

Embryologists can observe the behavior of a spermatozoon by means of a microscope. It resembles a translucent tadpole since it has a long lashing tail and a circular head. The shape of the tail expedites the spermatozoon to progress keenly after it is evacuated from the reproductive gland. The tail supports propulsion of the spermatozoon towards the uterus in pursuit of an egg in the salpinges. Moreover, the tail of the spermatozoon enables the required motion to bind to and further penetrate a mature oocyte when it arrives.

Male human infertility or subfertility occurs when male reproductive cells fail to let a fertile female conceive a child or delay pregnancy after one or more years of regular unprotected sexual intercourse [2,3]. When a man fails to produce an adequate quantity of spermatozoa and/or produces low quality spermatozoa, these spermatozoa are called sub-optimal.

The generation of low quality spermatozoa minimizes the pregnancy rate [4]. These spermatozoa can be immotile or/and abnormal in shape. The immotile spermatozoa cannot move up to the fallopian tubes. As a result, they cannot fertilize a female ovum. The abnormally shaped spermatozoa may be able to travel, but even if they manage to reach the female gametocyte, they may not bind to and penetrate its shell and therefore the woman may reduce her chance of getting pregnant. Conversely, when a male body produces a low number of reproductive cells, the probability that one of the sperm in the semen unites an egg to form a zygote significantly decreases. There are some possible factors in the male body such as the age, anxiety, pathogens, and diet, which may impact the number of abnormal sperm in the semen [5,6]. It is clear that high sperm head deformities lead to low fertilization, implantation, and pregnancy rates [7].

Human infertility is a disease of the reproductive system that affects more than 10% of couples across the globe and over 30% of reported cases are related to men [8]. The crucial step for male fertility diagnosis relies on the examination of sperm morphology through the seminogram. The key types of defects of the abnormal sperm are: head, neck, tail and excess residual cytoplasm [9], but head abnormalities play a major role in male infertility. There are two main tasks in sperm morphology analysis; the first is to classify the types of defects in the sperm head, neck, and tail, and the second is to estimate the number of abnormal sperm. In this study, we emphasis on the classification of the head morphological defects or abnormalities.

In practice, the results derived from manual morphological analyses of sperm rely heavily on the expertise of laboratory technicians [10]. Moreover, this manual examination is laborious, non-repeatable, time intensive, and there is a high degree of inter and intra-laboratory variability [11]. For animal spermatozoon analysis, there exist certain computer-aided sperm analysis (CASA) using commercial software. However, human semen samples have a much lower quality of spermatozoa than animal semen samples [12], and thus the same software may not be directly applied to human spermatozoon analysis. Furthermore, it was found that the application of the CASA system to analyze human spermatozoa required human assistance which may affect results of the assessment subjectively [13].

According to the above analysis, it is important to design accurate, automatic, and efficient artificial intelligence (AI) systems to improve the numerical analysis of human spermatozoa from the sperm images. Actually, the morphological classification of human sperm heads plays an important role in the numerical analysis of human spermatozoa, which has already attracted extensive interest relating to the diagnosis of male infertility. Our main interest here is to focus on the development of deep learning model to extract features directly from sperm images for morphological classification of human sperm heads. According to the World Health Organization (WHO), there are 11 abnormal categories of human sperm heads, which are defined according to certain particular morphometric characteristics of the heads. They differ in shape, size, and texture in a very complicated way so that the task becomes extremely difficult even for a human expert. In addition to intra-class differences, there are also inter-class similarities. For instance, an elongated Amorphous head is similar to a Tapered head or pear-shaped like Pyriform head, and a Tapered head that is constricted near the tail is identical to a Pyriform head.

From the public SCIAN dataset [14] and recent studies, it was found that the morphological classification of human sperm heads is very challenging for the following reasons: (1) There is a high degree of inter-class similarities as well as certain intra-class differences in some cases; (2) Low-magnification microscopic images of sperm heads are very noisy; (3) The size of the images is very small: the length and width of the sperm heads are about 4 µm and 3 µm, respectively, and the size of each image is approximately 35 by 35 pixels; (4) The number of sperm head examples is insufficient for training a complex machine learning model; (5) The two-thirds of the examples in the SCIAN (partial agreement) dataset consists of only 2-out-of-3 human expert agreement; (6) The classes are highly imbalanced (e.g., the Amorphous class has ten times more examples than the Small class); (7) The Amorphous class has no common structure, and their forms can change in different ways.

The main aim of this research is to develop, implement, and calibrate an advanced deep learning model in the context of morphological sperm assessment. This specialized deep CNN architecture can accurately classify microscopic human sperm head images according to WHO criteria. Our proposed deep learning architecture is good to expedite the automatic classification process of human sperm heads. This innovative method has the potential of deep learning to exceed embryologists in terms of accuracy, reliability, and throughput.

## 2. Related Work

According to the guidelines of WHO, there are 11 categories of abnormalities of human sperm heads: Tapered, Pyriform, Amorphous, Small, Small acrosome, Large, Large acrosome, Round, Two heads, Vacuolated, and Vacuoles in the post-acrosomal region. Among them, the Tapered, Pyriform, Amorphous, and including Normal categories can mainly be discriminated by the precise shapes of their samples. Therefore, it is extremely challenging to distinguish them even by an embryologist. However, the remaining abnormal categories can mainly be discriminated by the different sizes of their heads or the existence of vacuoles or the acrosome and thus it is relatively easy to distinguish and recognize them. For sperm classification tasks, conventional machine learning algorithms have been adopted to alleviate the laborious work of embryologists and improve classification performance. Nonetheless, the input of these algorithms contain certain manually extracted spermatozoon features like the head perimeter, area, and eccentricity [15,16]. Although several approaches have been established for the semen analysis of animals (e.g., [17,18]), there are only a few approaches for the morphological classification of human sperm heads. We now briefly review some machine learning approaches related to the morphological classification of human sperm heads.

In 2017, Chang et al. [14] introduced a gold standard dataset, SCIAN-MorphoSpermGS, for the analysis and evaluation of morphological classification of human sperm heads. Notably, there had been no open and free available dataset before this gold standard dataset became public. The SCIAN dataset has five classes of human sperm heads for semen analysis namely: Normal, Tapered, Pyriform, Amorphous, and Small, which are available in the WHO laboratory manual. It consists of 1854 sperm head images, which were labeled by three Chilean referent domain experts as specified by the guidelines of WHO. Chang et al. [19] further proposed a two-phase analysis pipeline, CE-SVM, for the morphological classification of human sperm heads in the SCIAN dataset. In the first phase, a classifier is trained to distinguish the Amorphous category from the remaining four categories. In the second phase, four classifiers are trained for the four non-Amorphous categories, where each classifier aims to distinguish the specific non-Amorphous category from the Amorphous category.

From a different direction, Shaker et al. [20] released the Human Sperm Head Morphology (HuSHeM) dataset and proposed an adaptive dictionary learning (APDL)-based approach, which extracts certain square patches from the sperm head images to train the dictionaries to recognize those sperm head categories. At the evaluation stage, square patches are recreated with the dictionary and the minimum overall error among those of all the categories is computed to identify the best sperm head category. Recently, with the fast development of deep learning techniques, Riordon et al. [21] used a VGG16 architecture (FT-VGG) for the morphological classification of human sperm heads. First, the VGG network was pre-trained on ImageNet [22] and then fine-tuned on the SCIAN dataset. Their experimental results demonstrated that this automatic deep learning method can facilitate and boost the seminogram effectively.

## 3. Methodology

### 3.1. Datasets Descrption, Partitioning, and Augmentation

**Figure 2:**
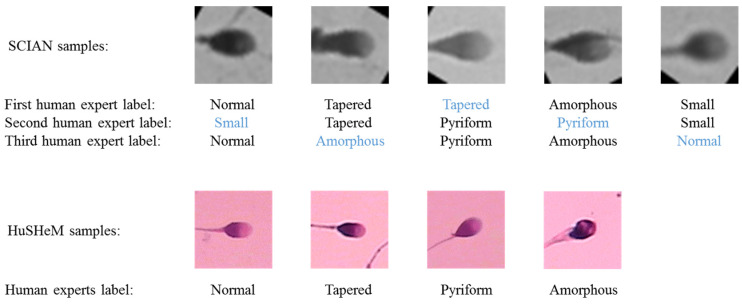
Typical samples of human sperm heads of microscopic images of the five classes in the partial agreement setting of the SCIAN dataset and the four classes of the HuSHeM dataset.

SCIAN [14] is a gold-standard dataset for the morphological classification of human sperm heads with five categories: Normal, Tapered, Pyriform, Amorphous, and Small. The manual labeling of sperm head images in this dataset was independently performed by three referent Chilean experts who had experience in sperm morphology examination for several years. The images in this dataset are of greyscale with stained sperm heads, being taken at 63× magnification and their height and width are both 35 pixels or 7 µm. There are three separate agreement settings among three domain experts: no agreement, partial agreement, and total agreement. The first set consists of 1854 sperm head images (175 Normal, 420 Tapered, 188 Pyriform, 919 Amorphous, and 152 Small), but an image in this set can be labeled manually into three dissimilar classes by three domain experts. The second set comprises 1132 images (100 Normal, 228 Tapered, 76 Pyriform, 656 Amorphous, and 72 Small) but an image can be labeled into two different sperm head classes. The third set includes 384 images (35 Normal, 69 Tapered, 7 Pyriform, 262 Amorphous, and 11 Small), all three experts assigned the same class label to a sperm head image. From the number of images in these three sets, we can appreciate the difficulty of the morphological classification of human sperm heads even by human experts. For illustration (Figure 2), we show typical samples of human sperm heads of microscopic images of the five classes in the partial agreement setting of the SCIAN dataset and the four classes of the HuSHeM dataset.

For effective usage of the SCIAN dataset, all images are converted into three channels and rotated so that all human sperm heads share the same orientation. For the convenience of comparison, we also adopt a stratified five-fold cross-validation scheme as used in [21]. That is, the SCIAN dataset is randomly partitioned into five parts, where the four parts that contain approximately 80% of the data from each class form the training set, while the remaining part which has roughly 20% of the data from each class forms the test set. The complete training/evaluation procedure is repeated five times for all possible choices of the training and test sets and the average results is reported. To compare the performance of our proposed model directly with the previous published results [20,21], each five-fold cross-validation procedure runs three times for stability. In addition, 20% of the fold-1 images are considered as the development set to tune the hyperparameters of our proposed network (see Table 1 for the details).

In order to tackle the issue of skewed classes and training image scarcity, we implement more augmentation options to the minority classes, and less augmentation options to the majority classes to balance the sample size in each class of the training set. Therefore, the training set is extended virtually for the deep learning task with the actual classes being balanced. For example, the Pyriform and Amorphous classes in the fold-5 partition of the partial agreement setting (see Table 1) have 61 and 524 distinct images, respectively, but the sample image sizes in the two corresponding augmented classes are similar, i.e., 6283 and 6288, respectively.

As for the specific data augmentation, we adopt three common techniques for the SCIAN dataset: rotation, translation, and flipping. For each sample image, we rotate it by −5 to 5 degrees. For translation, we shift ~6% of the original image to the left, the right, up, and down. For flipping, we vertically flip the image. For both partial (Table 1) and total (Table 2) agreement settings, we make a stratified five-fold partition of the SCIAN dataset as well as its augmentation for the evaluation of the proposed deep architecture. Similar pre-processing, partitioning, and augmentation are performed on the HuSHeM dataset. The details are available in Section 4.3 and Table 3. It should be noted that our data augmentation options are only implemented for the training set, while the development and test sets only contain the original sample images.

### 3.2. Proposed Deep CNN Architecture and Learning Paradigm

**Figure 3:**
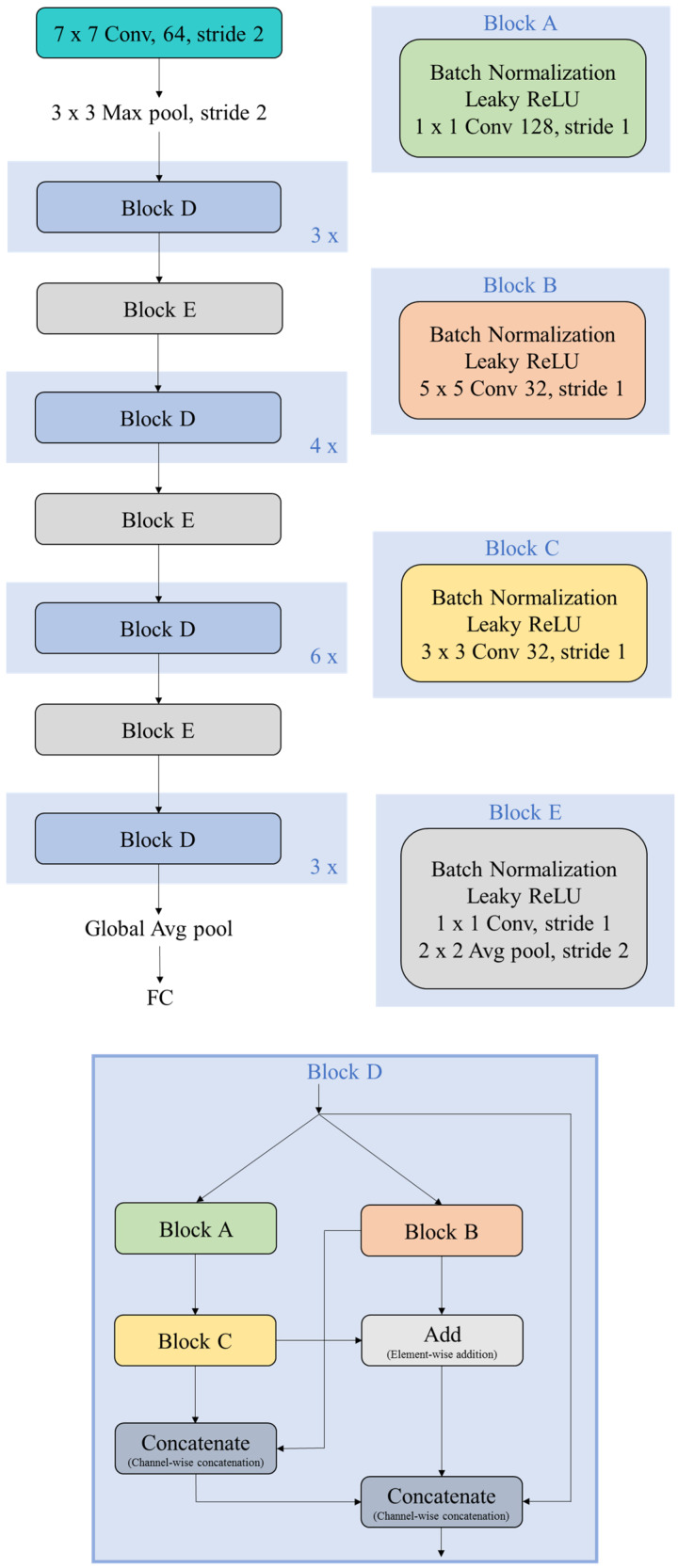
The layout of the proposed deep CNN architecture, where ‘FC’ denotes the fully connected layer.

With the above pre-processing, partitioning, and augmentation of the SCIAN and the HuSHeM datasets, we try to design a deep CNN architecture especially for the morphological classification of human sperm heads. The deep CNN architectures [23,24,25,26,27,28] obtained the top results in many complicated classification and regression tasks. Since the morphological classification of human sperm heads is an image classification task, it is proper to apply the deep CNN to solve such a complicated problem. To combat this problem, our proposed deep CNN architecture, Morphological Classification of Human Sperm Heads (MC-HSH), consists of four main kernel components as shown in Figure 3.

Specifically, components one to four are all denoted by Block D with 3, 4, 6, and 3 repetitions from top to bottom in the upper left subfigure, respectively. It is clear that ‘x’ with prefix 3, 4, or 6 near the lower right corner of Block D denote that this block repeats 3, 4, or 6 times. Moreover, these components are connected by Block E each time. Actually, Block D is a combination of Block A, B, and C, and their concatenation and addition operations are shown in the bottom subfigure, where Block A, B, and C are shown in the upper right. The numbers of filters in Block A, B, and C are 128, 32, and 32, while their filter sizes are 1 by 1, 5 by 5, and 3 by 3, respectively. In the first component, we use 9 convolutional layers to detect the simple features such as those of nucleus and nuclear vacuoles. In the second component, we use 12 convolutional layers to detect the complex features such as the acrosome and outer acrosome membrane patterns. In the third component, we further implement 18 convolutional layers to identify the more complex features such as those of peri and sub-acrosomal space. In the fourth component, we add 9 more convolutional layers to learn the features that are quite precise to describe the categories of human sperm heads. As a result, this deep CNN architecture is effective for the morphological classification of human sperm heads.

There are a total of 53 convolutional layers in our proposed deep CNN architecture. Before each convolutional layer, the batch normalization [29] and LeakyReLU [30] are implemented. In Block D, we use element-wise addition and channel-wise concatenation to make this architecture more effective for this classification. The number of filters in Block E is equal to half the number of existing channels. LeCun uniform initializers [31] are used to initialize the weights and biases. LeakyReLU and softmax are utilized as the activation functions for the convolutional layers and output layer, respectively. We use an L_2_ norm as the kernel regularizer with λ being 0.005 in a dense layer to prevent overfitting.

We utilize the Adam learning algorithm [32] to train our proposed deep CNN model with a mini batch size of 1024 for 50 epochs for the SCIAN dataset. The learning rate is set by 0.0005 with a 0.0055 decay rate, while β_1_ and β_2_ are respectively set to be 0.9 and 0.999 in the moment estimates. Moreover, the categorical cross entropy is employed as the loss/cost function. We implement the training procedure by using Keras [33] with TensorFlow [34] backend on GPU. We further tune the hyperparameters of the model on the development set. Specifically, the hyperparameters are selected according to the lowest loss of the model evaluated on the development set. Finally, the obtained model is used to assess the test set.

## 4. Experimental Results

In this section, we ran five-fold cross-validation analyses for our proposed deep CNN model for the morphological classification of human sperm heads in the SCIAN and the HuSHeM datasets. We tested it on both partial and total agreement settings of the SCIAN and the HuSHeM, and compared our results with the state-of-the-art methods. We used the metrics of the precision, recall, specificity, F_1_-score, Jaccard similarity coefficient, geometric mean (G-mean), Matthews correlation coefficient (MCC), and Cohen’s kappa score (CKS) for the classification assessment and comparison. There are two types of averaging: macro-averaging and weighted-averaging. That is, when computing the average of the indices of the classes, equal weight is assigned to all the classes in the way of macro-averaging, while a different weight is assigned to a class that is proportional to the number of its images in the way of weighted-averaging. According to the stratified five-fold partitions of the SCIAN (in both partial and total agreement settings) and the HuSHeM datasets and the learning paradigm given in the previous section, we implement our model using the TensorFlow and Keras framework on a NVIDIA GeForce GTX 1080 card with 8GB GDDR5X memory. The training process takes roughly 18 hours in total for the SCIAN dataset. We also evaluate our deep learning model on the HuSHeM dataset. The training process takes approximately 5 hours in total on this dataset. In the following subsections, we summarize and discuss the experimental results and comparisons in both partial and total agreement settings of the SCIAN dataset as well as the results on the HuSHeM dataset.

### 4.1. On the Stratified Five-Fold Partition of the SCIAN Dataset with the Partial Agreement Setting

**Figure 4:**
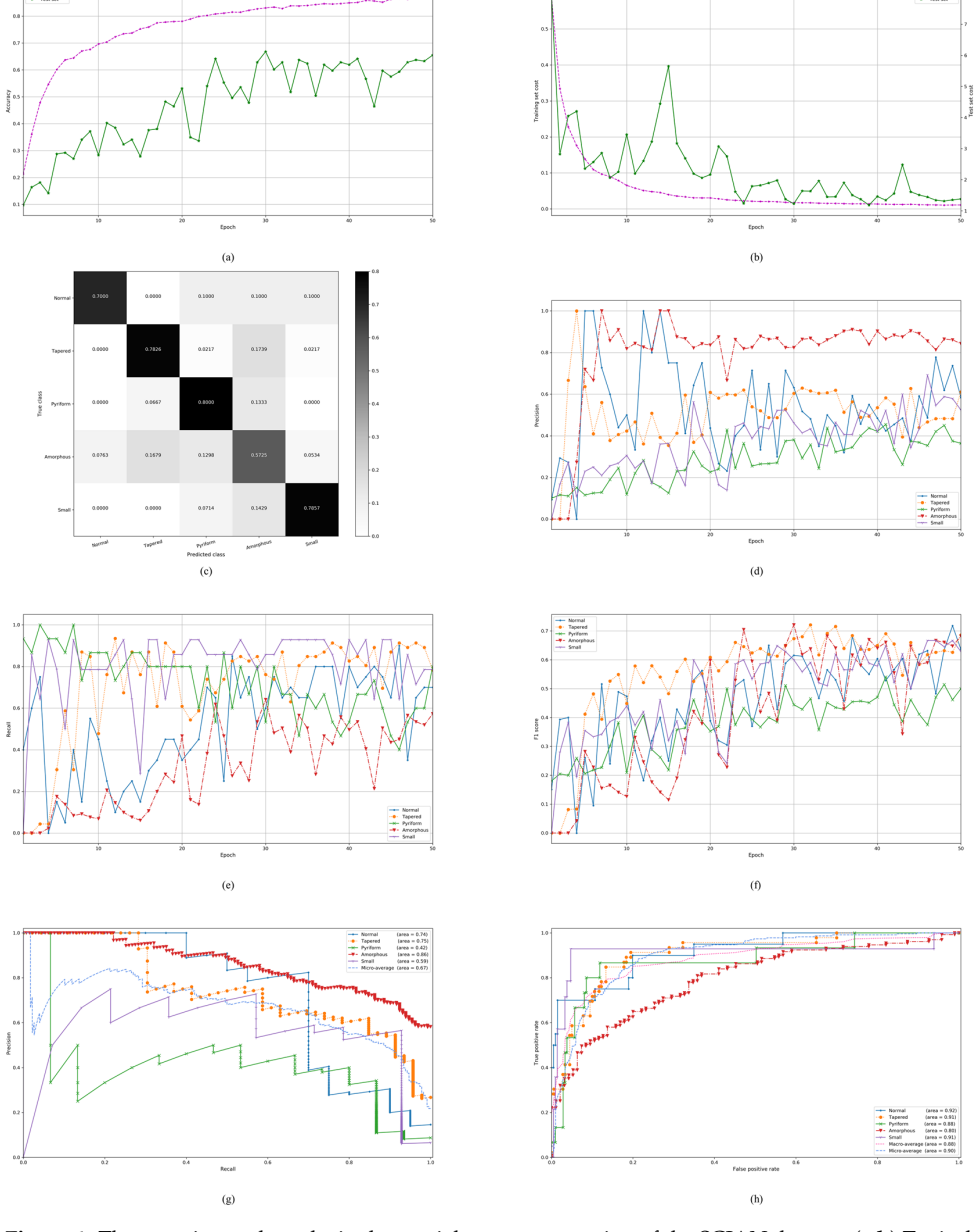
The experimental results in the partial agreement setting of the SCIAN dataset: (**a**,**b**) Typical classification accuracy and cost curves with the number of epochs during the training on the training and test sets; (**c**) The confusion matrix on the test set; (**d**–**f**) The precision, recall, and F_1_-score curves of five classes respectively on the test set; (**g**) The precision-recall curves of five classes on the test set as well as their micro-averaging precision-recall curve; (**h**) The receiver operating characteristic (ROC) curves of five classes on the test set as well as their macro and micro-averaging ROC curves.

Our proposed model is first evaluated on the stratified five-fold partition of the SCIAN dataset in the partial agreement setting. We train the deep CNN architecture on each choice of training set in the partial agreement setting and tune the hyperparameters on the development set. The experimental results of our proposed model on the SCIAN dataset with the partial agreement setting is shown in Figure 4a–h. The detailed experimental results are shown in Supplementary Materials (Figures S1–S8). Figure 4a–b show typical classification accuracy and cost curves with the number of epochs on a specific choice of training and test sets. It is seen that the training process converged within 50 epochs. Notably, our proposed model achieves much better accuracy and recall than the previous methods in the partial agreement setting (Table 4). By the stratified five-fold cross-validation, we get the confusion matrix (Figure 4c), from which we can see how often images of each individual class (Normal, Tapered, Pyriform, Amorphous, and Small) are predicted by our proposed model on the test set in the partial agreement setting only for a typical run. We also get the average confusion matrix over 15 runs (5 folds × 3 runs) as shown in Table 5. After carefully examining these tables, we find that the Amorphous class is very difficult to distinguish from the remaining classes. The main reason for this may be that the Amorphous class has a variety of forms. On the contrary, we also find that the average true positive rate (TPR) of the Tapered class is relatively high so that the Tapered images can be easily detected. The precision, recall, and F_1_-score curves of five classes respectively on the test set in the partial agreement setting through a typical run are shown by Figure 4d–f. From these three subfigures, we can see that the five class curves for each of the precision, recall and F_1_-score globally tend to stabilize and increase as the number of epochs increase. We further plot the precision-recall curves of five classes on the test set as well as their micro-averaging precision-recall curve (Figure 4g) for a typical run. A large area under the precision-recall curve (PR-AUC) signifies the high precision as well as the high recall. Having observed this subfigure, we find out that the Amorphous class has the highest PR-AUC, whereas the Pyriform class has the lowest one. Furthermore, we plot the receiver operating characteristic (ROC) curves of five classes on the test set as well as their macro and micro-averaging ROC curves (Figure 4h) for a typical run. The area under the ROC curve (ROC-AUC) is also valuable because it shows the tradeoff between the TPR and false positive rate (FPR). From this subfigure, we can further find out that the Normal class has the highest ROC-AUC, whereas the Amorphous class has the lowest one. Finally, we summarize the detailed results of each fold in the partial agreement setting for each run in Table 6 which includes all the possible evaluation metrics such as the precision, recall, specificity, F_1_-score, Jaccard similarity coefficient, G-mean, ROC-AUC, PR-AUC, MCC, CKS, and evaluation time. The standard deviation in the last row of this table shows the stability of the result of our proposed model with a training run for each index. Since all standard deviations are less than 0.09, our proposed model is therefore quite stable with the learning algorithm.

### 4.2. On the Stratified Five-Fold Partition of the SCIAN Dataset with the Total Agreement Setting

**Figure 5:**
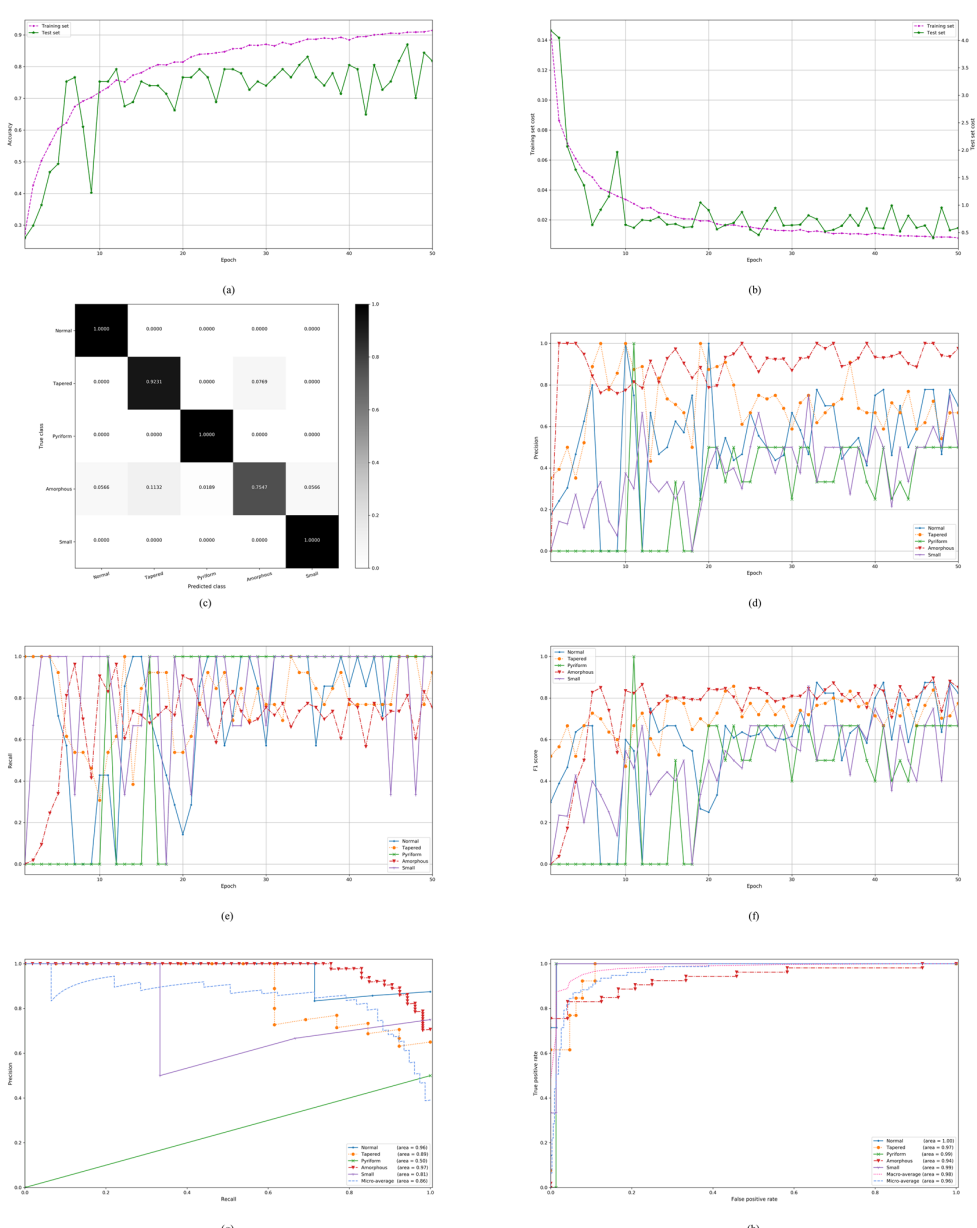
The experimental results in the total agreement setting of the SCIAN dataset: (**a**,**b**) Typical classification accuracy and cost curves with the number of epochs during the training on the training and test sets; (**c**) The confusion matrix on the test set; (**d**–**f**) The precision, recall, and F_1_-score curves of five classes respectively the on test set; (**g**) The precision-recall curves of five classes on the test set as well as their micro-averaging precision-recall curve; (**h**) The receiver operating characteristic (ROC) curves of five classes on the test set as well as their macro and micro-averaging ROC curves.

Our proposed model is further evaluated on the stratified five-fold partition of the SCIAN dataset in the total agreement setting. Similarly, we train the deep CNN architecture on each choice of training set in the total agreement setting. However, we no longer tune the hyperparameters since they have been tuned in the previous case of the partial agreement setting. The experimental results of our proposed model in the total agreement setting are shown in Figure 5a–h. The detailed experimental evaluations in the total agreement setting are available in Supplementary Materials (Figures S9–S16). Specifically, Figure 5a–b show typical classification accuracy and cost curves during the training on a specific choice of training and test sets. It is seen that our proposed model obtains very high classification accuracy in the total agreement setting when the training process converged. Our proposed model also attains a much higher accuracy and recall than the previous methods in the total agreement setting, which is clearly shown in Table 7 by simply comparing the precision, specificity, and F_1_-score indices of our proposed model and the VGG model in [21]. For the elaborate comparisons with the models in [20,21], we employ the stratified five-fold cross-validation scheme in the total agreement setting. Figure 5c illustrates the confusion matrix of the classification for a typical run. We also compute the average confusion matrix over 15 runs (5 folds × 3 runs), as shown in Table 8. According to these tables, the Amorphous class remains the most difficult class to be differentiated from the other classes. Nevertheless, we can also find that the average TPRs of the Normal, Tapered, Pyriform, and Small classes become better. Therefore, the experimental results confirm that the Amorphous class is the most difficult to distinguish from the other classes. The precision, recall, and F_1_-score curves of five classes on the test set in the total agreement setting through a typical run are shown in Figure 5d–f, respectively. From these three subfigures, we can again see that the five class curves of each of the precision, recall, and F_1_-score globally tend to stabilize and increase as the number of epochs increases. We further plot the precision-recall curves of five classes on the test set as well as their micro-averaging precision-recall curve in Figure 5g for a typical run. It is clearly observed from this subfigure that the Amorphous and Normal classes have a higher PR-AUC than the other classes, while the Pyriform class has the lowest one. Moreover, we plot the ROC curves of five classes on the test set as well as their macro and micro-averaging ROC curves in Figure 5h for a typical run. From this subfigure, we can see that the Normal class has the highest ROC-AUC, while the Amorphous class has the lowest one. The detailed results of each fold in the total agreement setting for each run are available in Table 9. From the last row of this table, we can also see low standard deviations of different indices from our proposed model with a training run in the total agreement setting, demonstrating the stability of our proposed model with the learning algorithm. As the agreement is strict in this case, common and essential features can be extracted effectively from the labeled images so that the classification results are improved considerably. In summary, our proposed model attains an overall accuracy of 77%, a macro precision of 64%, a macro recall of 88%, and a macro specificity of 94% in the total agreement setting, which are much better than the previous results.

### 4.3. On the Stratified Five-Fold Partition of the HuSHeM Dataset

Our proposed model is finally evaluated on the HuSHeM [20] dataset. This is another dataset for the morphological classification of human sperm heads with 216 images (54 Normal, 53 Tapered, 57 Pyriform, and 52 Amorphous). Its images are also manually annotated by three human experts, but only the images with three-expert agreement are recorded. Each image consists of 131 by 131 pixels, being taken at 100× magnification.

In the pre-processing step, we first rotate the images so that all human sperm heads share the same orientation. We then crop the sample images so that the sperm heads appear in the center of the images. After this step, the images are reduced to 90 by 90 pixels. Approximately 80% of the images are considered for training and the remaining images for the evaluation. We further employ data augmentation techniques to solve the scarcity of training images. As for the data augmentation, we adopt three common techniques as we used in the training set of the SCIAN dataset. For rotation, we rotate the training image by −25 to 25 degrees. For translation, we shift ~6% of the original image to the left, the right, up, and down. For flipping, we vertically flip the image. Due to the same distribution of classes within this dataset, we apply equal augmentation options to each class. For the convenience of comparison, we also adopt a stratified five-fold cross-validation scheme as used in [20,21]. We utilize the Adam learning algorithm to train our proposed deep CNN model with mini a batch size of 256 for 25 epochs for the HuSHeM dataset. To compare the performance of our proposed model directly with previously published results [20,21], each five-fold cross-validation procedure runs three times for stability.

**Figure 6:**
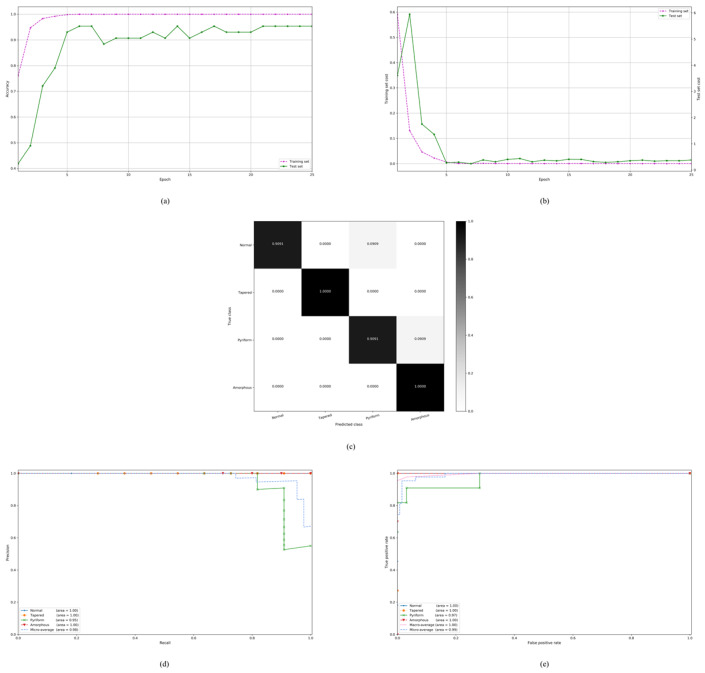
The experimental results of the HuSHeM dataset: (**a**,**b**) Typical classification accuracy and cost curves with the number of epochs during the training on the training and test sets; (**c**) The confusion matrix on the test set; (**d**) The precision-recall curves of four classes on the test set as well as their micro-averaging precision-recall curve; (**e**) The receiver operating characteristic (ROC) curves of four classes on the test set as well as their macro and micro-averaging ROC curves.

The experimental results of our proposed model on the HuSHeM dataset are shown in Figure 6a–e. The detailed experimental results are shown in Supplementary Materials (Figures S17–S21). The experimental results of our proposed model as well as the previous methods on the HuSHeM dataset are shown in Table 10. It is clearly seen that our proposed model achieves better accuracy, recall, precision, specificity, and F_1_-score than previous methods. Moreover, from the confusion matrix of our proposed model (Table 11), we can see that Pyriform classes in the test set are predicted 97% correctly. Results on the HuSHeM dataset are the average of 15 runs (5 folds × 3 runs). We also plot the precision-recall curves of four classes on the test set as well as their micro-averaging precision-recall curve in Figure 6d for a typical run. Furthermore, we plot the ROC curves of four classes on the test set as well as their macro and micro-averaging ROC curves in Figure 6e for a typical run. Finally, we summarize the detailed results of each fold for each run in Table 12 which includes all the possible evaluation metrics such as the precision, recall, specificity, F_1_-score, Jaccard, G-mean, ROC-AUC, PR-AUC, MCC, CKS, and evaluation time.

## 5. Discussion and Conclusions

We have established an advanced deep CNN architecture, MC-HSH, specially for the morphological classification of human sperm heads. In this deep learning architecture, there are a total of 53 convolutional layers. Before each convolutional layer, the batch normalization and LeakyReLU are used. We also apply the channel-wise concatenation and element-wise addition to make this model more effective for the morphological classification of human sperm heads. We employ the L_2_ penalty as the kernel regularizer in the dense layer to prevent overfitting. We utilize several layers and multiple filter sizes, but fewer filters and parameters, and we also make a new arrangement of convolutional layers, addition and concatenation operations for this classification task.

According to the WHO criteria [9], human sperm heads are classified into categories such as Normal, Tapered, Pyriform, Amorphous, and Small and their morphological classification is very challenging. Based on a golden standard SCIAN dataset of microscopic sperm images and the HuSHeM dataset, data-driven machine learning models and algorithms can be utilized to solve this difficult problem. By making careful pre-processing, partition, and argumentation of the SCIAN and the HuSHeM datasets, we design a specialized deep CNN architecture for the morphological classification of sperm heads based on the microscopic human sperm head images. The stratified five-fold cross-validation results demonstrate that our proposed model (along with the deep learning algorithm) is much more effective than the previous methods [14,19,20,21] for the morphological classification of human sperm heads. The performance indices on five classes (see Table 4, Table 5, Table 7 and Table 8) indicate that it is reliable in recognizing the images in the Normal class as well as the four abnormal classes. By attaining the embryologist level performance of the classification, our proposed model is also a balanced classifier where the TPR is similar to the positive predictive value (PPV).

It can be found from Table 4 and Table 7 that the previous methods are not so powerful to extract effective features from microscopic images for the classification of human sperm heads. Our proposed model achieves 68% and 88% average TPR on the SCIAN dataset in the partial and total agreement settings, respectively. We find out that our proposed model improves the accuracy and recall by a factor of 29% and 10%, respectively, in the partial agreement setting and 46% and 22%, respectively, in the total agreement setting compared with the state-of-the-art results reported in [21]. In the total agreement setting, our proposed model achieves a much better accuracy (77%) and recall (88%) because the training set has more images and the test set has the total expert agreement images in comparison with the accuracy (63%) and recall (68%) of the partial agreement setting. Our proposed model can extract the morphometric features for seminogram which are significant for sperm binding to the oocyte. The morphological classification of human sperm heads is an intricate problem because of intrinsic inter-class similarities and intra-class variabilities. Our proposed model achieves better classification results than the previous state-of-the-art methods without using transfer leaning. On the HuSHeM dataset, the results of our proposed model are also better than the state-of-the-art results. Our proposed approach achieves 96% accuracy and 95% recall on the HuSHeM dataset. The accuracy, recall, precision, and F_1_-score increase approximately 2%, whereas the specificity improves roughly 0.5% in comparison with [21]. The results of our proposed model are much better on the HuSHeM dataset than the SCIAN dataset. This improvement is due to three main reasons: (1) the HuSHeM dataset has only four sperm head classes; (2) its images have a high resolution; (3) and all of its images are 3-out-of-3 human expert agreement. The evaluation time of our proposed model is ~0.2 milliseconds (ms) for the SCIAN dataset, while ~0.9 ms for the HuSHeM dataset per image.

Developing an automated classification system of human sperm heads can greatly reduce the workload of embryologists and also decrease the subjectivity and inaccuracy of the classification induced by the human error. This automated system can become necessary and more valuable when experienced embryologists are not readily available and for inexperienced clinicians in underdeveloped countries. In fact, the classification results of our proposed model are comparable to those of the domain experts. Consequently, our proposed model can even be used to assign a class label to any new sperm head image, and this deep CNN architecture is good to expedite the automatic classification procedure of human sperm heads. Indeed, our research provides more strong evidence that the deep learning approach is able to play a key role in healthcare systems, assisting doctors to achieve higher conception and gestation rates. Our proposed architecture shows the potential of deep learning to surpass embryologists in terms of throughput, accuracy and reliability.

It is worth indicating the limitations of this study. As mentioned before, experiments are conducted on two publicly available datasets. The SCIAN dataset has 1132 and 384 human sperm heads images in the partial and total agreement settings, respectively, while the HuSHeM dataset has only 216 human sperm head images. These numbers of images are relatively small. Consequently, to obtain better generalizability, it is essential to increase the number of images for experimentation in the future. Secondly, due to limited computational power and memory the training time is high. Lastly, additional work remains to be done to evaluate the deep learning models in fertility clinics.

## Figures and Tables

**Table 1:** The stratified five-fold partition of the SCIAN dataset (partial agreement), where the numbers denote the distinct sample sizes in different classes while the numbers in parentheses denote the total number of augmented with the addition of original samples in different classes at each fold. Moreover, the bold numbers in the training set (fold-1) denote the number of samples in different classes assigned to the development set for tuning the hyperparameters of the network. To avoid repetition, folds 2 and 3 are described together.

Fold	Set	Sperm Head Classes					Total
		Normal	Tapered	Pyriform	Amorphous	Small	
1	Train	80–20 (4860)	182–46 (4896)	60–15 (4860)	525–131 (4728)	58–14 (4840)	905–226 (24184)
	Test	20	46	16	131	14	227
2 and 3	Train	80 (6400)	182 (6370)	61 (6405)	525 (6300)	58 (6380)	906 (31855)
	Test	20	46	15	131	14	226
4	Train	80 (6240)	183 (6222)	61 (6283)	525 (6300)	57 (6270)	906 (31315)
	Test	20	45	15	131	15	226
5	Train	80 (6240)	183 (6222)	61 (6283)	524 (6288)	57 (6270)	905 (31303)
	Test	20	45	15	132	15	227

**Table 2:** The stratified five-fold partition of the SCIAN dataset (total agreement), where the numbers denote the distinct sample sizes in different classes while the numbers in parentheses denote the total number of augmented with the addition of original samples in different classes at each fold. To avoid repetition, folds 1 and 2 are described together.

Fold	Set	Sperm Head Classes					Total
		Normal	Tapered	Pyriform	Amorphous	Small	
1 and 2	Train	93 (7719)	214 (7704)	74 (7696)	604 (7852)	70 (7700)	1055 (38671)
	Test	7	14	2	52	2	77
3	Train	93 (7719)	214 (7704)	75 (7725)	604 (7852)	70 (7700)	1056 (38700)
	Test	7	14	1	52	2	76
4	Train	93 (7719)	214 (7704)	75 (7725)	603 (7839)	70 (7700)	1055 (38687)
	Test	7	14	1	53	2	77
5	Train	93 (7626)	215 (7525)	75 (7575)	603 (7839)	69 (7590)	1055 (38155)
	Test	7	13	1	53	3	77

**Table 3:** The stratified five-fold partition of the HuSHeM dataset, where the numbers denote the distinct sample sizes in different classes while the numbers in parentheses denote the total number of augmented with the addition of original samples in different classes at each fold. To avoid repetition, folds 1, 2 and 3 are described together.

Fold	Set	Sperm Head Classes				Total
		Normal	Tapered	Pyriform	Amorphous	
1, 2 and 3	Train	43 (4730)	42 (4620)	46 (5060)	42 (4620)	173 (19030)
	Test	11	11	11	10	43
4	Train	43 (4730)	43 (4730)	45 (4950)	41 (4510)	172 (18920)
	Test	11	10	12	11	44
5	Train	44 (4840)	43 (4730)	45 (4950)	41 (4510)	173 (19030)
	Test	10	10	12	11	43

**Table 4:** The performance comparison of our proposed model with the previous methods in the partial agreement setting of the SCIAN dataset in terms of accuracy, precision, recall, specificity, and F_1_-score metrics. Bold font shows the best results. All the metrics are described in percentages. The accuracy, precision, specificity, and F_1_-score of the method in [21] were not reported directly, but calculated from its confusion matrix. The symbol ‘-’ stands for unreported results.

Model	True Positive Rate					Accuracy (Weighted Average TPR)	Recall (Average TPR)	Precision (Macro)	Specificity (Macro)	F_1_-Score (Macro)
	Normal	Tapered	Pyriform	Amorphous	Small					
MorphoSpermGS (SVM with Zernike moments) [14]	44	62	33	23	70	36	46	-	-	-
MorphoSpermGS (SVM with Fourier descriptors) [14]	57	68	53	15	54	34	49	-	-	-
CE-SVM [19]	62	64	50	30	**82**	44	58	-	-	-
APDL [20]	**71**	67	**71**	35	68	49	62	-	-	-
FT-VGG [21]	67	57	69	38	78	49	62	47	87	53
Proposed model (MC-HSH)	70	**79**	62	**57**	71	**63**	**68**	**56**	**90**	**61**

**Table 5:** The average confusion matrix on the stratified five-fold partition of the SCIAN dataset in the partial agreement setting, where each cell value (in percent) is the average of 15 runs (5 folds × 3 runs).

**True Class**	**Normal**	**70**	3	3	20	4
	**Tapered**	2	**79**	5	13	1
	**Pyriform**	3	8	**62**	26	1
	**Amorphous**	10	16	8	**57**	9
	**Small**	10	2	1	16	**71**
		**Normal**	**Tapered**	**Pyriform**	**Amorphous**	**Small**
		**Predicted Class**				

**Table 6:** The stratified five-fold cross-validation results for the morphological classification of human sperm heads in the partial agreement setting of the SCIAN dataset for every fold and every run, where all the metrics except Matthews correlation coefficient (MCC) and Cohen’s kappa score (CKS) are described in percentages.

Fold	Run	Precision		Recall		Specificity		F_1_-Score		Jaccard		G-mean		ROC-AUC		PR-AUC	MCC	CKS	Evaluation Time per Image (milliseconds)
		Macro	Weighted	Macro	Weighted	Macro	Weighted	Macro	Weighted	Macro	Weighted	Macro	Weighted	Macro	Micro	Micro			
					Accuracy														
1	First	57	71	67	67	90	86	60	67	44	51	77	75	88	90	69	+0.52	+0.50	~0.2
	Second	54	70	67	63	90	87	58	64	41	48	77	74	87	89	63	+0.48	+0.47	
	Third	53	70	66	63	90	87	56	64	40	46	76	74	87	89	64	+0.48	+0.46	
2	First	59	72	73	65	90	88	63	66	47	49	81	75	89	90	69	+0.52	+0.50	
	Second	58	72	73	65	90	87	63	66	46	50	81	76	88	90	67	+0.52	+0.50	
	Third	62	72	71	67	91	86	65	68	48	51	80	76	89	91	72	+0.53	+0.52	
3	First	50	66	63	56	88	86	52	56	36	39	73	68	85	84	53	+0.43	+0.39	
	Second	50	68	63	54	88	88	52	55	35	38	74	68	84	84	51	+0.42	+0.38	
	Third	47	63	62	52	87	85	50	53	34	36	73	66	83	83	48	+0.38	+0.35	
4	First	58	71	69	65	90	86	62	66	45	49	79	75	89	91	72	+0.51	+0.49	
	Second	62	72	69	69	91	84	65	70	48	54	79	76	89	91	73	+0.54	+0.53	
	Third	63	73	68	70	91	86	65	71	48	55	78	77	90	92	75	+0.55	+0.54	
5	First	56	70	68	65	90	85	60	66	43	50	78	74	88	89	63	+0.50	+0.48	
	Second	58	71	68	67	90	84	61	68	45	52	78	75	88	89	62	+0.51	+0.50	
	Third	55	70	69	63	90	86	59	64	42	47	79	73	87	87	58	+0.49	+0.47	
Average		56	70	68	63	90	86	59	64	43	48	78	73	87	89	64	+0.49	+0.47	
Standard deviation		0.0470	0.0263	0.0331	0.0533	0.0116	0.0122	0.0494	0.0539	0.0475	0.0572	0.0259	0.0336	0.0199	0.0282	0.0835	0.0478	0.0561	

**Table 7:** The performance comparison of our proposed model with the previous methods in the total agreement setting of the SCIAN dataset in terms of accuracy, precision, recall, specificity, and F_1_-score metrics. The bold font shows the best results. All the metrics are described in percentages. The accuracy, precision, specificity, and F_1_-score of the method in [21] were not reported directly, but calculated from its confusion matrix. The symbol ‘-’ stands for unreported results.

Model	True Positive Rate					Accuracy (Weighted Average TPR)	Recall (Average TPR)	Precision (Macro)	Specificity (Macro)	F_1_-Score (Macro)
	Normal	Tapered	Pyriform	Amorphous	Small					
CE-SVM [19]	74	70	92	30	**100**	46	73	-	-	-
FT-VGG [21]	72	67	95	44	84	53	72	45	90	55
Proposed model (MC-HSH)	**80**	**86**	**100**	**72**	**100**	**77**	**88**	**64**	**94**	**74**

**Table 8:** The average confusion matrix on the stratified five-fold partition of the SCIAN dataset in the total agreement setting, where each cell value (in percent) is the average of 15 runs (5 folds × 3 runs).

**True Class**	**Normal**	**80**	0	3	10	7
	**Tapered**	2	**86**	0	9	3
	**Pyriform**	0	0	**100**	0	0
	**Amorphous**	8	11	2	**72**	7
	**Small**	0	0	0	0	**100**
		**Normal**	**Tapered**	**Pyriform**	**Amorphous**	**Small**
		**Predicted Class**				

**Table 9:** The stratified five-fold cross-validation results for the morphological classification of human sperm heads in the total agreement setting of the SCIAN dataset for every fold and every run, where all the metrics except Matthews correlation coefficient (MCC) and Cohen’s kappa score (CKS) are described in percentages.

Fold	Run	Precision		Recall		Specificity		F_1_-Score		Jaccard		G-mean		ROC-AUC		PR-AUC	MCC	CKS
		Macro	Weighted	Macro	Weighted	Macro	Weighted	Macro	Weighted	Macro	Weighted	Macro	Weighted	Macro	Micro	Micro		
					Accuracy													
1	First	69	88	82	73	94	97	68	77	57	64	87	84	95	94	77	+0.61	+0.56
	Second	62	85	88	70	93	96	67	72	52	57	90	81	96	93	75	+0.60	+0.54
	Third	62	87	83	75	94	96	66	79	52	67	88	85	94	93	75	+0.62	+0.59
2	First	69	82	83	78	93	85	72	79	61	67	87	81	94	95	82	+0.61	+0. 60
	Second	60	84	87	77	93	90	65	79	50	66	89	83	94	94	81	+0.62	+0.60
	Third	62	80	83	77	92	94	68	77	52	64	87	80	95	95	85	+0.59	+0.58
3	First	62	83	91	76	92	91	71	77	56	63	92	83	95	94	77	+0.63	+0.60
	Second	64	84	87	80	93	87	72	81	57	69	90	83	95	94	79	+0.65	+0.64
	Third	57	84	89	74	94	94	64	75	49	61	91	83	94	93	73	+0.61	+0.57
4	First	60	87	89	79	95	95	67	81	52	69	92	87	97	96	86	+0.66	+0.64
	Second	70	81	85	77	92	86	75	78	63	65	89	81	95	95	82	+0.59	+0.58
	Third	66	86	91	79	95	92	74	80	59	68	92	85	97	95	82	+0.66	+0.64
5	First	59	86	92	77	94	95	68	78	53	64	93	85	97	96	84	+0.66	+0.62
	Second	67	89	94	79	95	97	75	80	61	68	94	87	98	96	86	+0.70	+0.66
	Third	67	87	94	82	96	95	76	83	61	71	94	88	98	96	86	+0.71	+0.69
Average		64	85	88	77	94	93	70	78	56	66	90	84	96	95	81	+ 0.63	+0.61
Standard deviation		0.0404	0.0261	0.0405	0.0300	0.0123	0.0401	0.0394	0.0267	0.0456	0.0356	0.0247	0.0243	0.0145	0.0112	0.0442	0.0374	0.0421

**Table 10:** The performance comparison of our proposed model with the previous methods on the HuSHeM dataset in terms of accuracy, recall, precision, specificity, and F_1_-score metrics. Bold font shows the best results. All the metrics are described in percentages. The specificity of the methods in [19,20,21] were not reported directly, but calculated from their confusion matrices.

Model	True Positive Rate				Accuracy	Recall	Precision	Specificity	F_1_-Score
	Normal	Tapered	Pyriform	Amorphous					
CE-SVM [19]	75.9	77.3	85.9	75.0	78.5	78.5	80.5	92.9	78.9
APDL [20]	94.4	94.3	87.7	94.2	92.2	92.3	93.5	97.5	92.9
FT-VGG [21]	**96.4**	**94.5**	92.3	93.2	94.0	94.1	94.7	98.1	94.1
Proposed model (MC-HSH)	95.8	**94.5**	**96.6**	**96.4**	**95.7**	**95.5**	**96.1**	**98.5**	**95.5**

**Table 11:** The average confusion matrix of our proposed model on the HuSHeM dataset, where each cell value (in percent) is the average of 15 runs.

**True Class**	**Normal**	**96**	3	1	0
	**Tapered**	1	**94**	2	3
	**Pyriform**	0	1	**97**	2
	**Amorphous**	2	2	0	**96**
		**Normal**	**Tapered**	**Pyriform**	**Amorphous**
		**Predicted Class**			

**Table 12:** The stratified five-fold cross-validation results for the morphological classification of human sperm heads on the HuSHeM dataset for every fold and every run, where all the metrics except Matthews correlation coefficient (MCC) and Cohen’s kappa score (CKS) are described in percentages.

Fold	Run	Precision		Recall		Specificity		F_1_-Score		Jaccard		G-mean		ROC-AUC		PR-AUC	MCC	CKS	Evaluation Timeper Image (milliseconds)
		Macro	Weighted	Macro	Weighted	Macro	Weighted	Macro	Weighted	Macro	Weighted	Macro	Weighted	Macro	Micro	Micro			
					Accuracy														
1	First	98	98	98	98	99	99	98	98	96	96	98	98	100	100	100	+0.97	+0.97	~0.9
	Second	100	100	100	100	100	100	100	100	100	100	100	100	100	100	100	+1.00	+1.00	
	Third	98	98	98	98	99	99	98	98	96	96	98	98	100	100	100	+0.97	+0.97	
2	First	95	96	95	95	98	98	95	95	91	91	97	97	100	99	98	+0.94	+0.94	
	Second	95	96	95	95	98	98	95	95	91	91	97	97	100	100	99	+0.94	+0.94	
	Third	93	93	93	93	98	98	93	93	87	87	95	95	100	99	99	+0.91	+0.91	
3	First	95	96	95	95	98	98	95	95	91	91	97	97	100	99	99	+0.94	+0.94	
	Second	95	96	95	95	98	98	95	95	91	91	97	97	99	99	98	+0.94	+0.94	
	Third	95	96	95	95	98	98	95	95	91	91	97	97	100	99	98	+0.94	+0.94	
4	First	98	98	98	98	99	99	98	98	95	96	98	98	100	100	100	+0.97	+0.97	
	Second	95	95	95	95	99	99	95	95	91	91	97	97	100	100	100	+0.94	+0.94	
	Third	95	96	95	95	99	99	95	95	91	92	96	97	100	100	100	+0.94	+0.94	
5	First	94	94	93	93	98	98	93	93	87	87	95	95	100	100	100	+0.91	+0.91	
	Second	95	96	96	95	99	99	95	95	91	91	97	97	100	99	98	+0.94	+0.94	
	Third	94	94	93	93	98	98	93	93	87	87	95	95	100	99	99	+0.91	+0.91	
Average		96	96	96	96	99	99	96	96	92	92	97	97	100	100	99	+0.94	+0.94	
Standard deviation		0.0191	0.0181	0.0207	0.0207	0.0064	0.0064	0.0207	0.0207	0.0365	0.0372	0.0133	0.0131	0.0026	0.0052	0.0086	0.0250	0.0250	

## Supplementary Materials

Available online at https://www.mdpi.com/2075-4418/10/5/325/s1. Figure S1. Detailed experimental results of the proposed model through 50 epochs in the partial agreement setting of the SCIAN dataset, where 15 classification accuracy curves with the number of epochs during the training on each of five possible choices of the training and test sets for 3 runs (5 folds × 3 runs) are illustrated; Figure S2. Detailed experimental results of the proposed model through 50 epochs in the partial agreement setting of the SCIAN dataset, where 15 cost curves with the number of epochs during the training on each of five possible choices of the training and test sets for 3 runs (5 folds × 3 runs) are illustrated; Figure S3. Detailed experimental results of the proposed model through 50 epochs in the partial agreement setting of the SCIAN dataset, where 15 confusion matrixes on each of five possible choices of the test sets for 3 runs (5 folds × 3 runs) are illustrated; Figure S4. Detailed experimental results of the proposed model through 50 epochs in the partial agreement setting of the SCIAN dataset, where 15 precision curves of each class with the number of epochs during the training on each of five possible choices of the test sets for 3 runs (5 folds × 3 runs) are illustrated; Figure S5. Detailed experimental results of the proposed model through 50 epochs in the partial agreement setting of the SCIAN dataset, where 15 recall curves of each class with the number of epochs during the training on each of five possible choices of the test sets for 3 runs (5 folds × 3 runs) are illustrated; Figure S6. Detailed experimental results of the proposed model through 50 epochs in the partial agreement setting of the SCIAN dataset, where 15 F1-score curves of each class with the number of epochs during the training on each of five possible choices of the test sets for 3 runs (5 folds × 3 runs) are illustrated; Figure S7. Detailed experimental results of proposed model in the partial agreement setting of the SCIAN dataset, where 15 precision-recall curves of each class and their micro-averaging precision-recall curve on each of five possible choices of the test sets for 3 runs (5 folds × 3 runs) are illustrated. A high area under the curve signifies the high precision as well as high recall; Figure S8. Detailed experimental results of proposed model in the partial agreement setting of the SCIAN dataset, where 15 receiver operating characteristic (ROC) curves of each class and their macro and micro-averaging ROC curves on each of five possible choices of the test sets for 3 runs (5 folds × 3 runs) are illustrated. These plots show the tradeoff between the true positive rate and false positive rate; Figure S9. Detailed experimental results of the proposed model through 50 epochs in the total agreement setting of the SCIAN dataset, where 15 classification accuracy curves with the number of epochs during the training on each of five possible choices of the training and test sets for 3 runs (5 folds × 3 runs) are illustrated; Figure S10. Detailed experimental results of the proposed model through 50 epochs in the total agreement setting of the SCIAN dataset, where 15 cost curves with the number of epochs during the training on each of five possible choices of the training and test sets for 3 runs (5 folds × 3 runs) are illustrated; Figure S11. Detailed experimental results of the proposed model through 50 epochs in the total agreement setting of the SCIAN dataset, where 15 confusion matrixes on each of five possible choices of the test sets for 3 runs (5 folds × 3 runs) are illustrated; Figure S12. Detailed experimental results of the proposed model through 50 epochs in the total agreement setting of the SCIAN dataset, where 15 precision curves of each class with the number of epochs during the training on each of five possible choices of the test sets for 3 runs (5 folds × 3 runs) are illustrated; Figure S13. Detailed experimental results of the proposed model through 50 epochs in the total agreement setting of the SCIAN dataset, where 15 recall curves of each class with the number of epochs during the training on each of five possible choices of the test sets for 3 runs (5 folds × 3 runs) are illustrated; Figure S14. Detailed experimental results of the proposed model through 50 epochs in the total agreement setting of the SCIAN dataset, where 15 F1-score curves of each class with the number of epochs during the training on each of five possible choices of the test sets for 3 runs (5 folds × 3 runs) are illustrated; Figure S15. Detailed experimental results of proposed model in the total agreement setting of the SCIAN dataset, where 15 precision-recall curves of each class and their micro-averaging precision-recall curve on each of five possible choices of the test sets for 3 runs (5 folds × 3 runs) are illustrated. A high area under the curve signifies the high precision as well as high recall; Figure S16. Detailed experimental results of proposed model in the total agreement setting of the SCIAN dataset, where 15 receiver operating characteristic (ROC) curves of each class and their macro and micro-averaging ROC curves on each of five possible choices of the test sets for 3 runs (5 folds × 3 runs) are illustrated. These plots show the tradeoff between the true positive rate and false positive rate; Figure S17. Detailed experimental results of the proposed model through 25 epochs of the HuSHeM dataset, where 15 classification accuracy curves with the number of epochs during the training on each of five possible choices of the training and test sets for 3 runs (5 folds × 3 runs) are illustrated; Figure S18. Detailed experimental results of the proposed model through 25 epochs of the HuSHeM dataset, where 15 cost curves with the number of epochs during the training on each of five possible choices of the training and test sets for 3 runs (5 folds × 3 runs) are illustrated; Figure S19. Detailed experimental results of the proposed model through 25 epochs of the HuSHeM dataset, where 15 confusion matrixes on each of five possible choices of the test sets for 3 runs (5 folds × 3 runs) are illustrated; Figure S20. Detailed experimental results of proposed model of the HuSHeM dataset, where 15 precision-recall curves of each class and their micro-averaging precision-recall curve on each of five possible choices of the test sets for 3 runs (5 folds × 3 runs) are illustrated. A high area under the curve signifies the high precision as well as high recall; Figure S21. Detailed experimental results of proposed model of the HuSHeM dataset, where 15 receiver operating characteristic (ROC) curves of each class and their macro and micro-averaging ROC curves on each of five possible choices of the test sets for 3 runs (5 folds × 3 runs) are illustrated. These plots show the tradeoff between the true positive rate and false positive rate.
