# Supplementary material for: Deep Learning-Based Morphological Classification of Human Sperm Heads
Source: Diagnostics (Basel). 2020 May 20;10(5):325. doi: 10.3390/diagnostics10050325 (PMC7277990; doi:10.3390/diagnostics10050325)
Supplement: Supplementary file 1 [file diagnostics-10-00325-s001.pdf]

Article

Deep Learning-Based Morphological Classification of Human Sperm Heads

Imran Iqbal [1], Ghulam Mustafa [2] and Jinwen Ma [1,*]

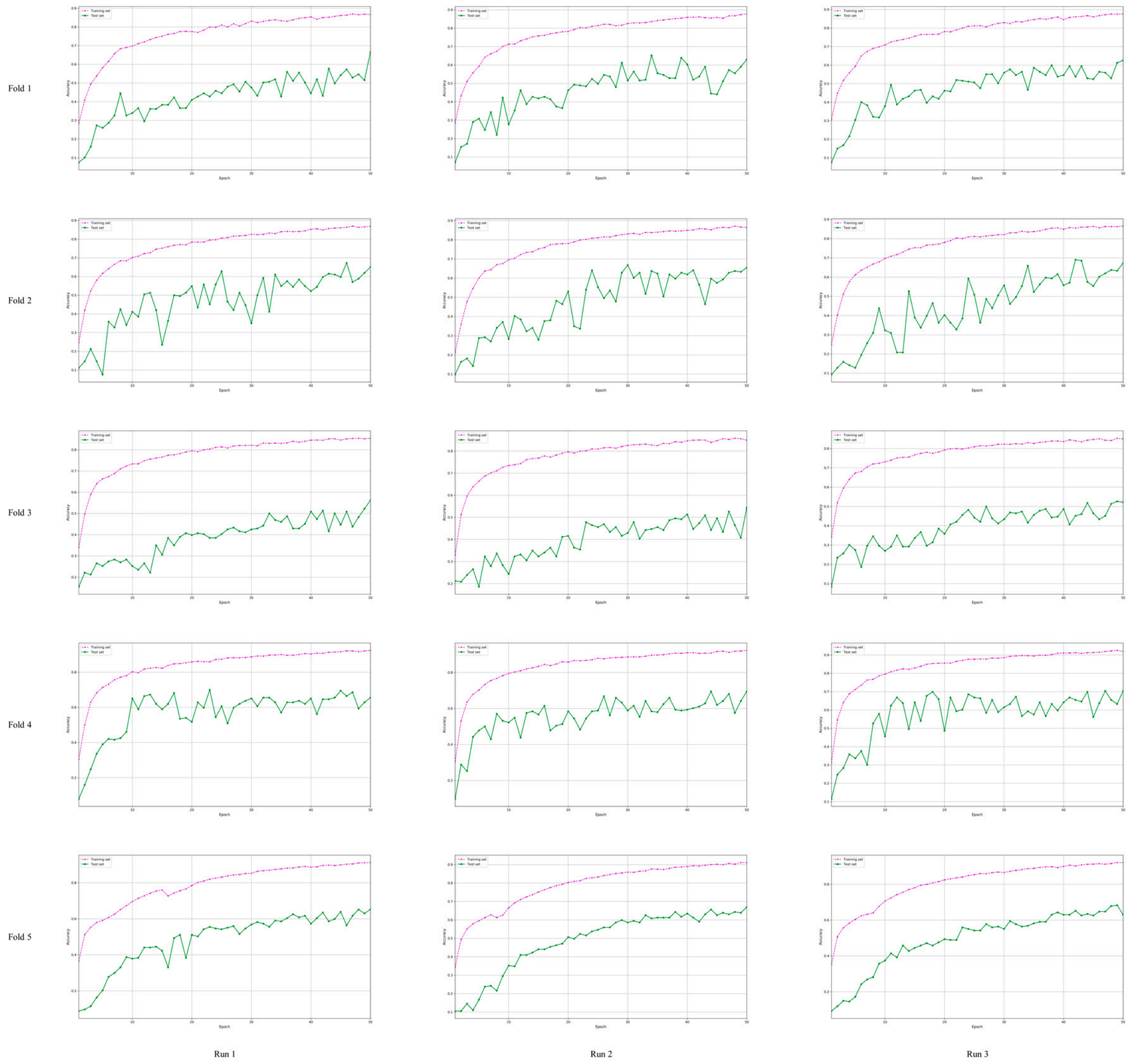

Figure S1. Detailed experimental results of the proposed model through 50 epochs in the partial agreement setting of the SCIAN dataset, where 15 classification accuracy curves with the number of epochs during the training on each of five possible choices of the training and test sets for 3 runs (5 folds × 3 runs) are illustrated.

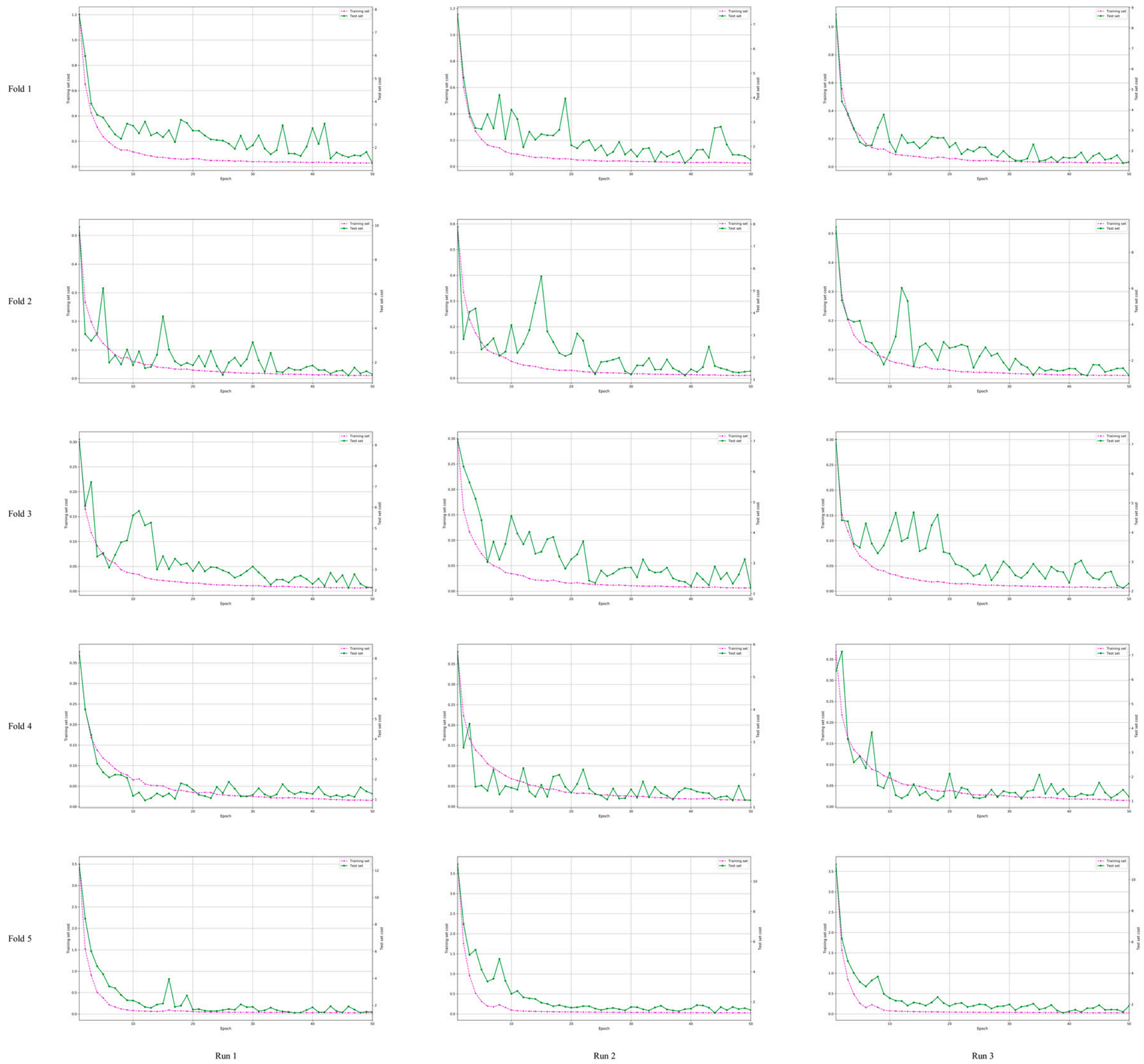

Figure S2. Detailed experimental results of the proposed model through 50 epochs in the partial agreement setting of the SCIAN dataset, where 15 cost curves with the number of epochs during the training on each of five possible choices of the training and test sets for 3 runs (5 folds × 3 runs) are illustrated.

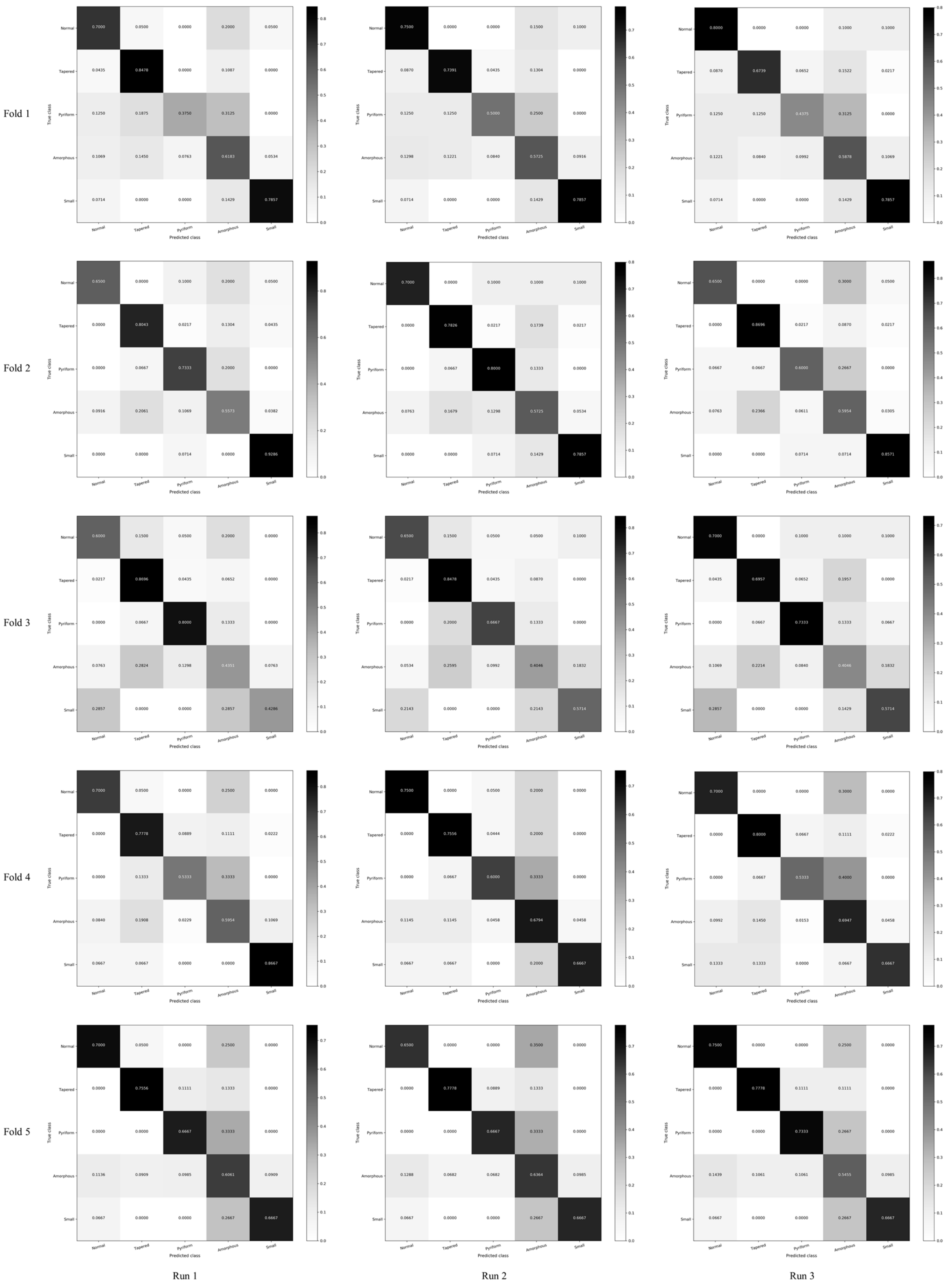

Figure S3. Detailed experimental results of the proposed model through 50 epochs in the partial agreement setting of the SCIAN dataset, where 15 confusion matrixes on each of five possible choices of the test sets for 3 runs (5 folds × 3 runs) are illustrated.

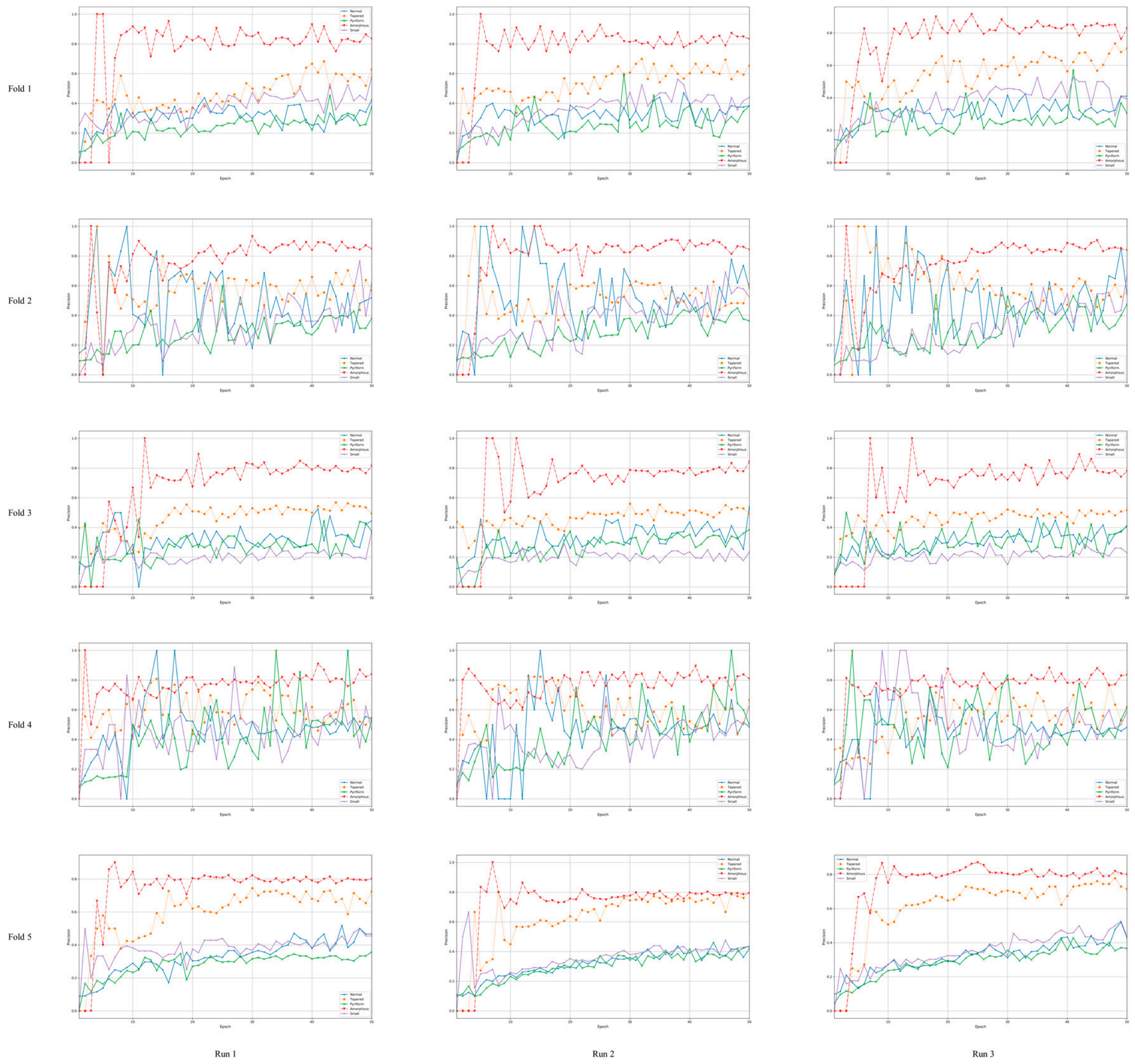

Figure S4. Detailed experimental results of the proposed model through 50 epochs in the partial agreement setting of the SCIAN dataset, where 15 precision curves of each class with the number of epochs during the training on each of five possible choices of the test sets for 3 runs (5 folds × 3 runs) are illustrated.

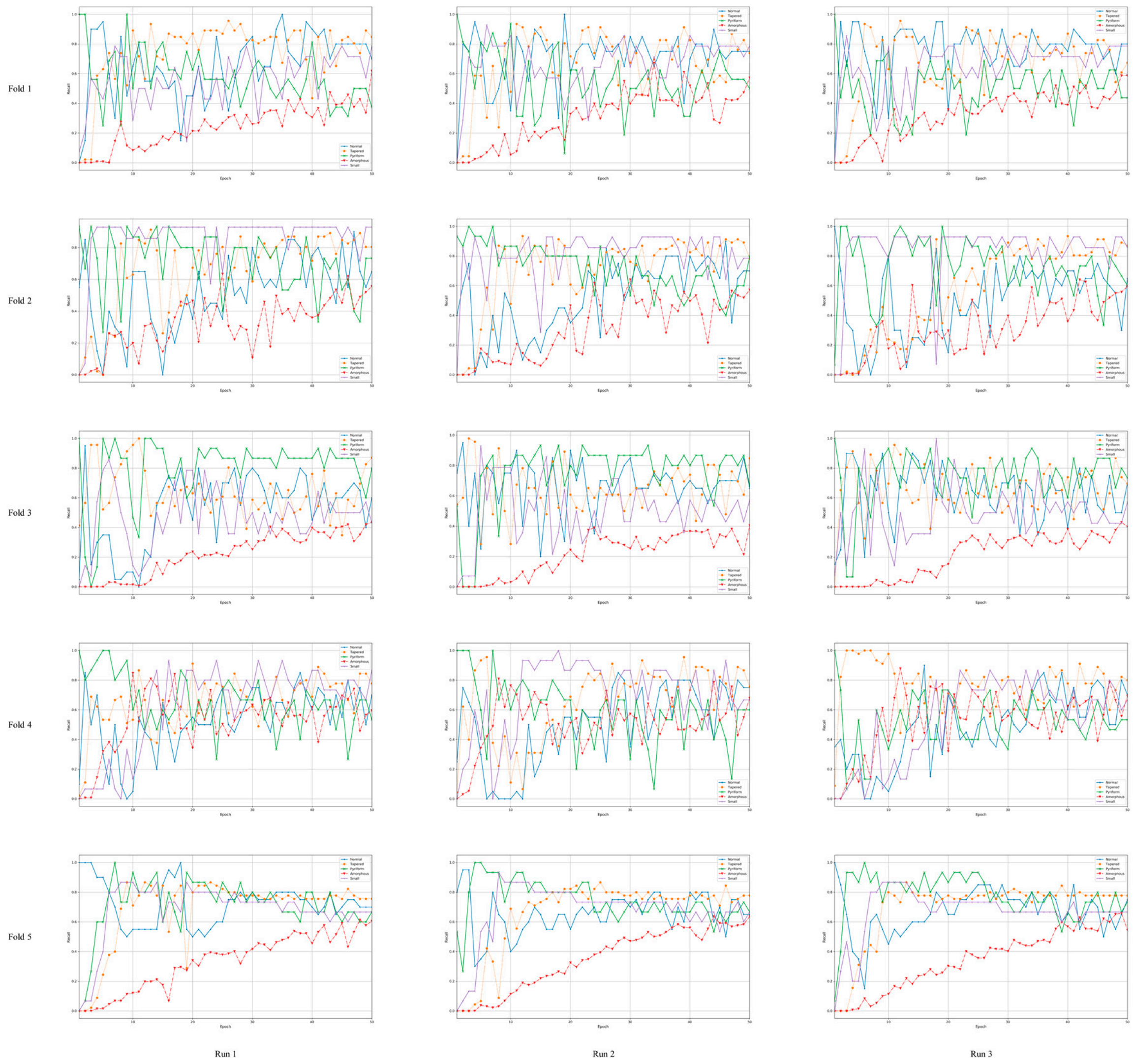

Figure S5. Detailed experimental results of the proposed model through 50 epochs in the partial agreement setting of the SCIAN dataset, where 15 recall curves of each class with the number of epochs during the training on each of five possible choices of the test sets for 3 runs (5 folds × 3 runs) are illustrated.

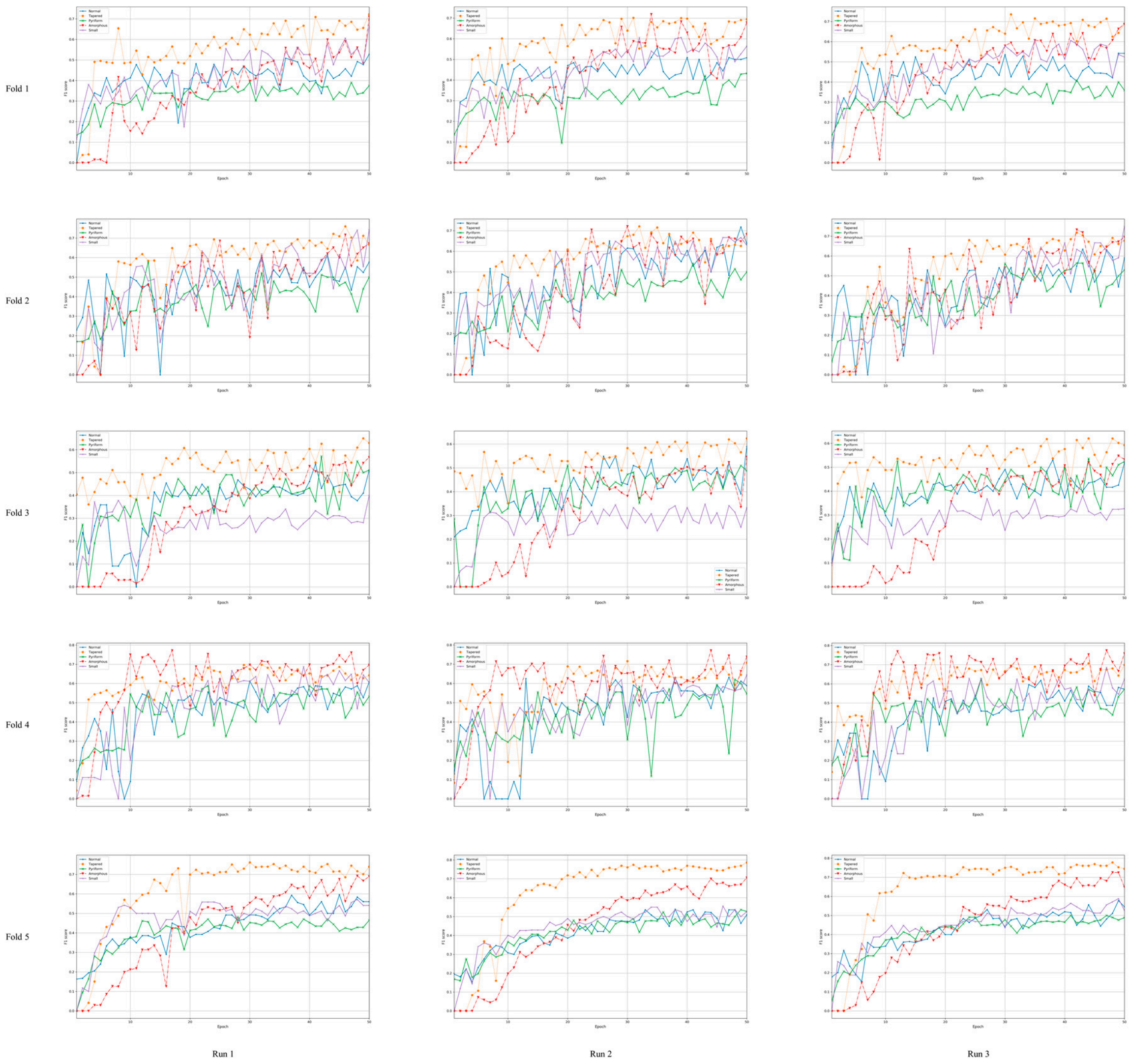

Figure S6. Detailed experimental results of the proposed model through 50 epochs in the partial agreement setting of the SCIAN dataset, where 15 F_1-score curves of each class with the number of epochs during the training on each of five possible choices of the test sets for 3 runs (5 folds × 3 runs) are illustrated.

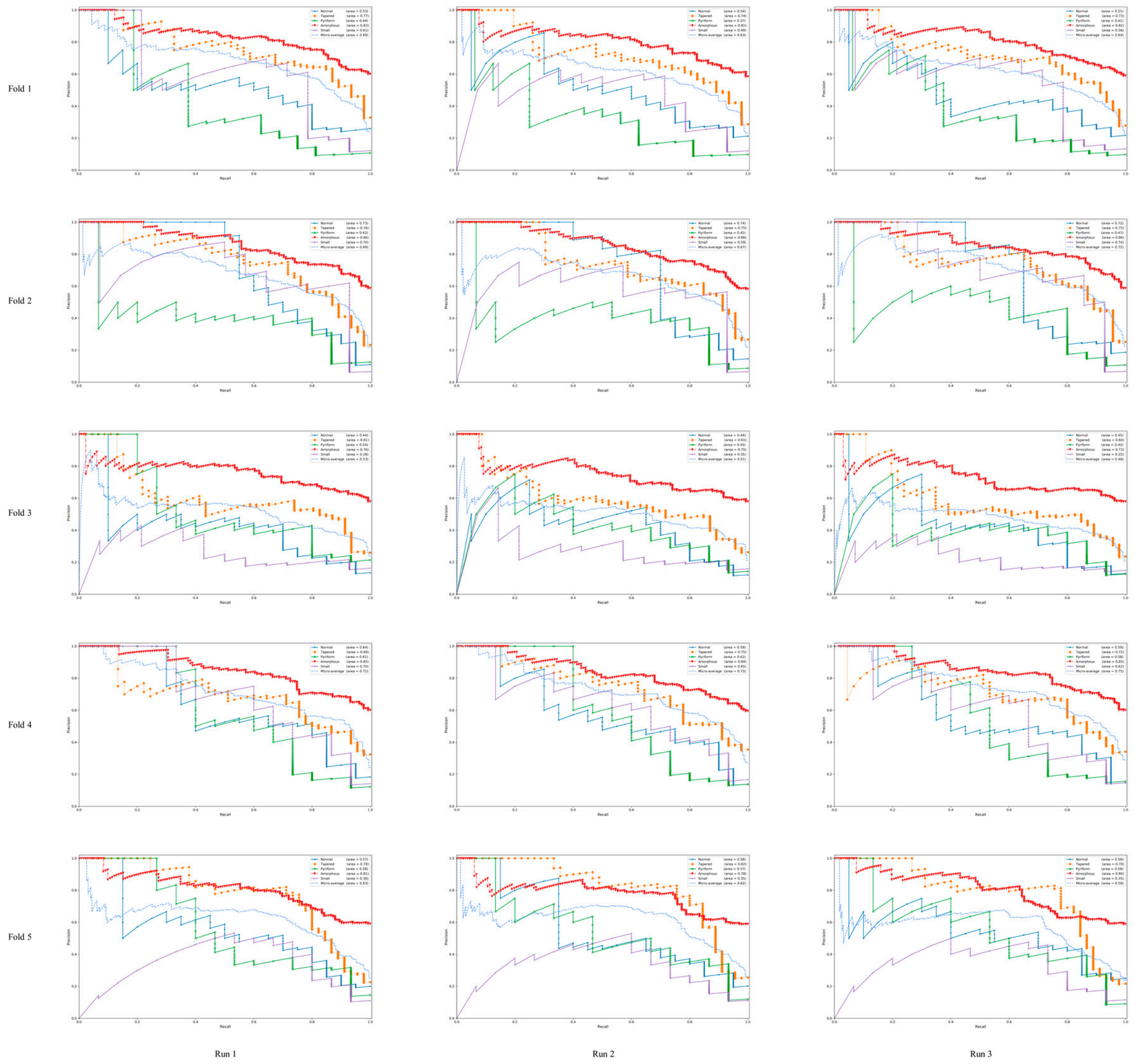

Figure S7. Detailed experimental results of proposed model in the partial agreement setting of the SCIAN dataset, where 15 precision-recall curves of each class and their micro-averaging precision-recall curve on each of five possible choices of the test sets for 3 runs (5 folds $\times$ 3 runs) are illustrated. A high area under the curve signifies the high precision as well as high recall.

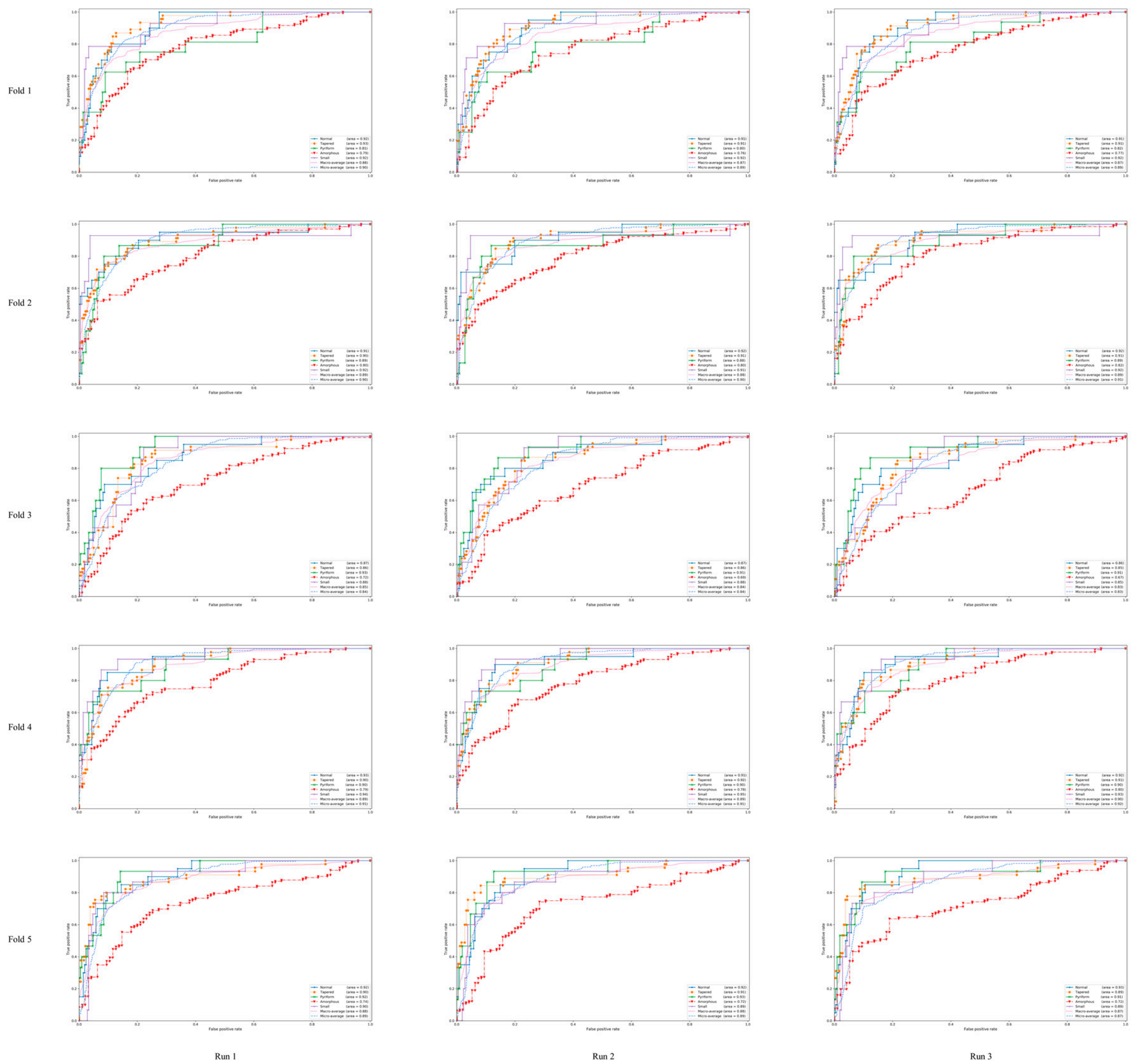

Figure S8. Detailed experimental results of proposed model in the partial agreement setting of the SCIAN dataset, where 15 receiver operating characteristic (ROC) curves of each class and their macro and micro-averaging ROC curves on each of five possible choices of the test sets for 3 runs (5 folds × 3 runs) are illustrated. These plots show the tradeoff between the true positive rate and false positive rate.

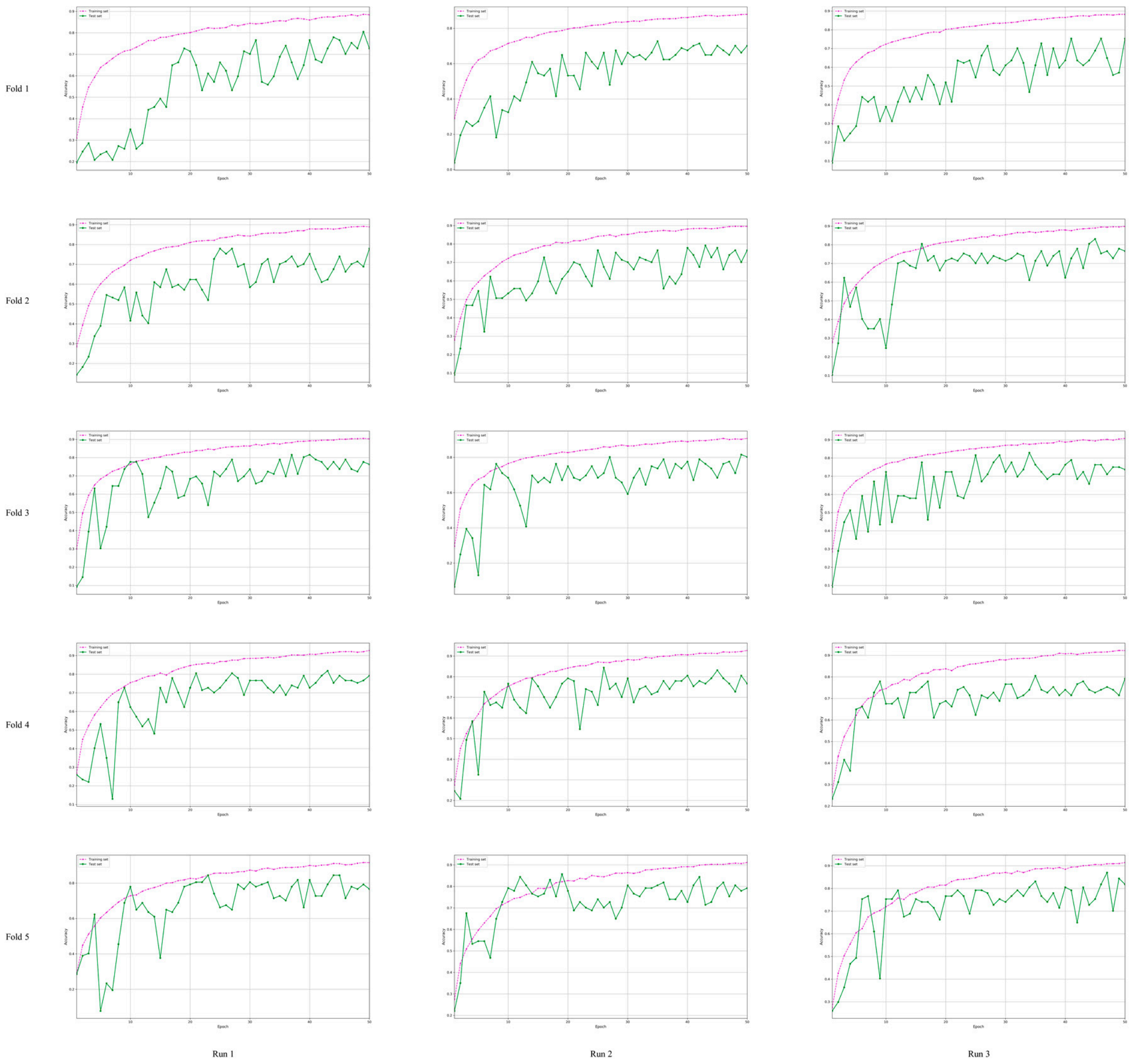

Figure S9. Detailed experimental results of the proposed model through 50 epochs in the total agreement setting of the SCIAN dataset, where 15 classification accuracy curves with the number of epochs during the training on each of five possible choices of the training and test sets for 3 runs (5 folds $\times$ 3 runs) are illustrated.

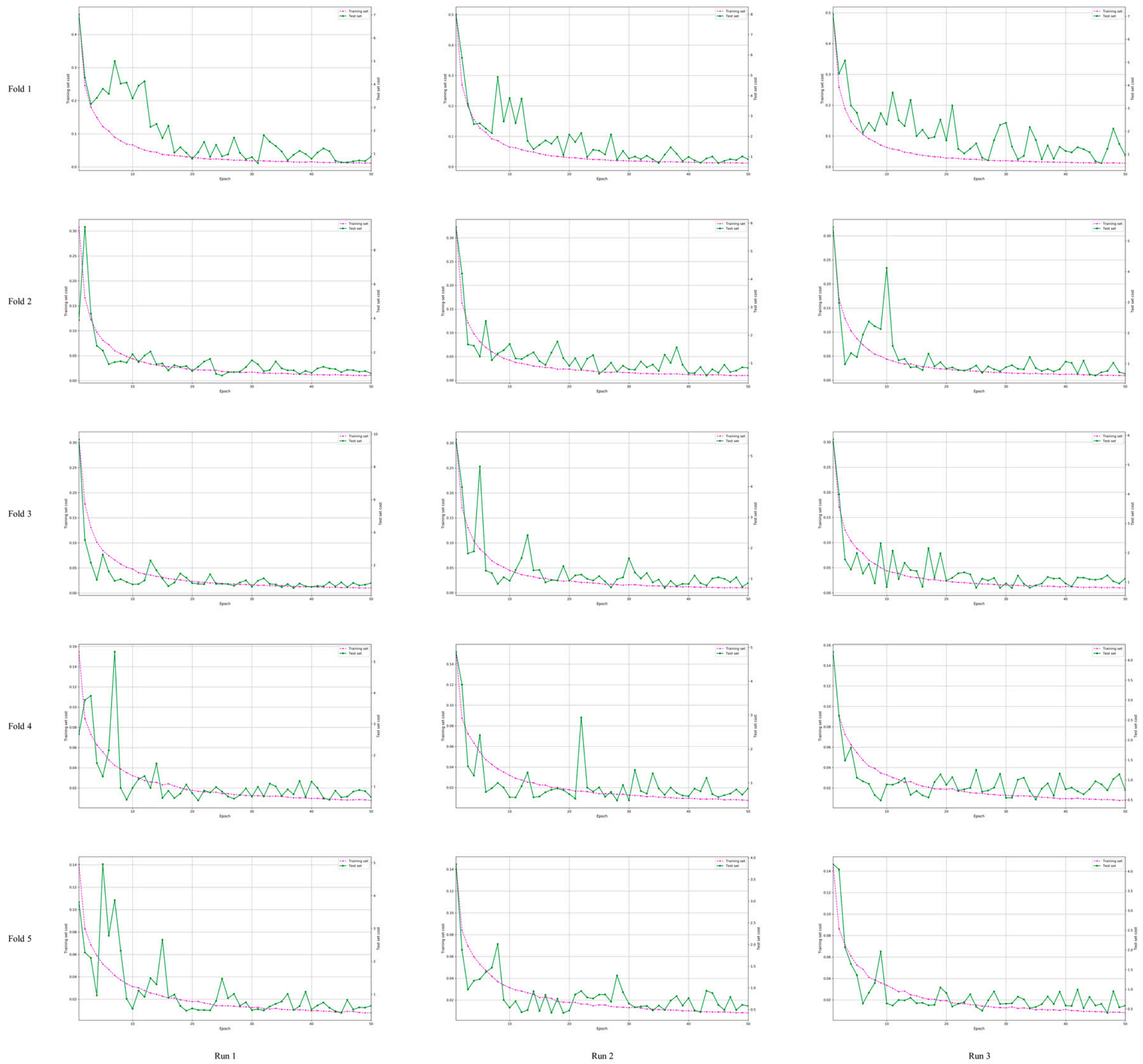

Figure S10. Detailed experimental results of the proposed model through 50 epochs in the total agreement setting of the SCIAN dataset, where 15 cost curves with the number of epochs during the training on each of five possible choices of the training and test sets for 3 runs (5 folds $\times$ 3 runs) are illustrated.

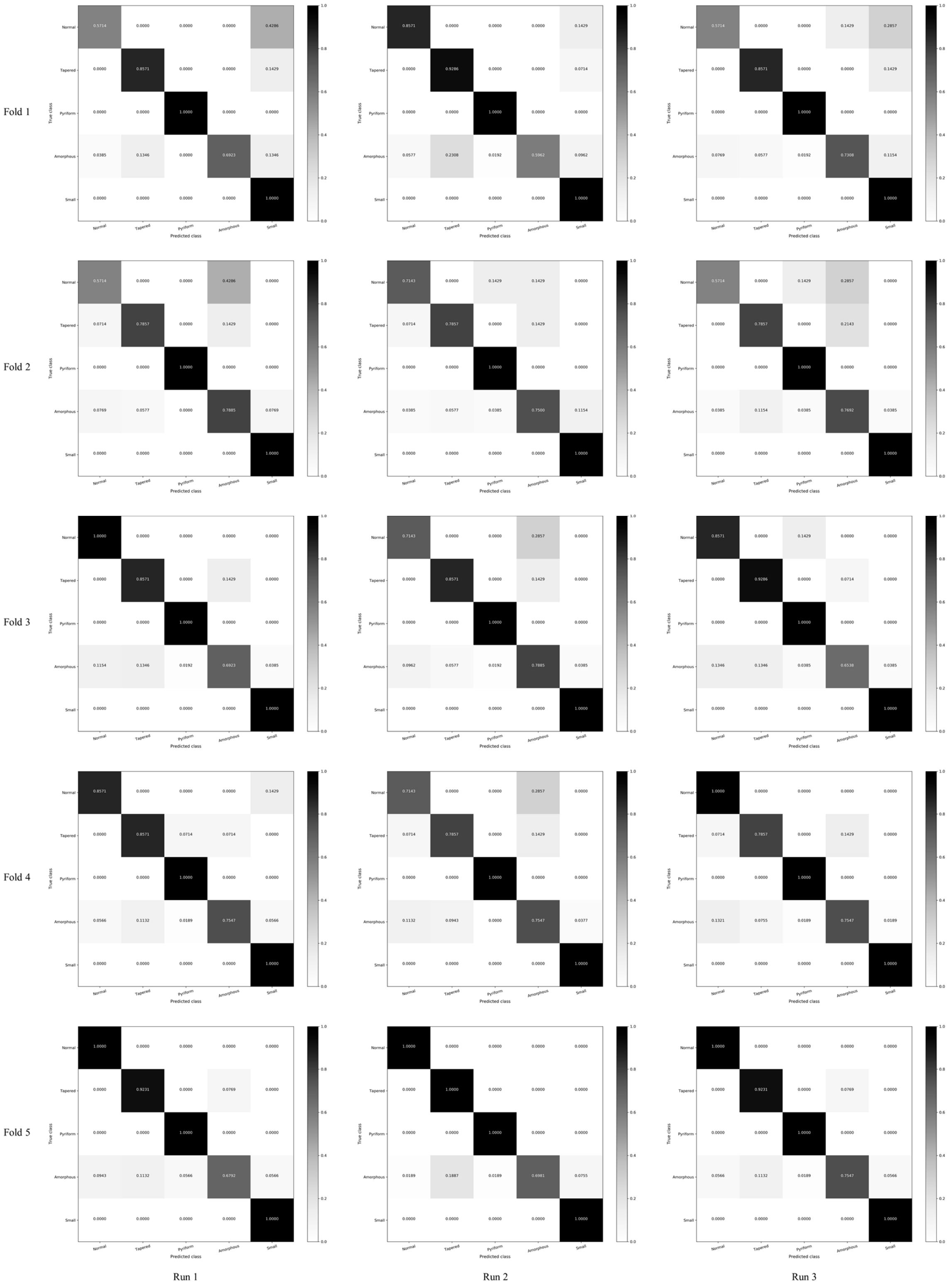

Figure S11. Detailed experimental results of the proposed model through 50 epochs in the total agreement setting of the SCIAN dataset, where 15 confusion matrixes on each of five possible choices of the test sets for 3 runs (5 folds $\times$ 3 runs) are illustrated.

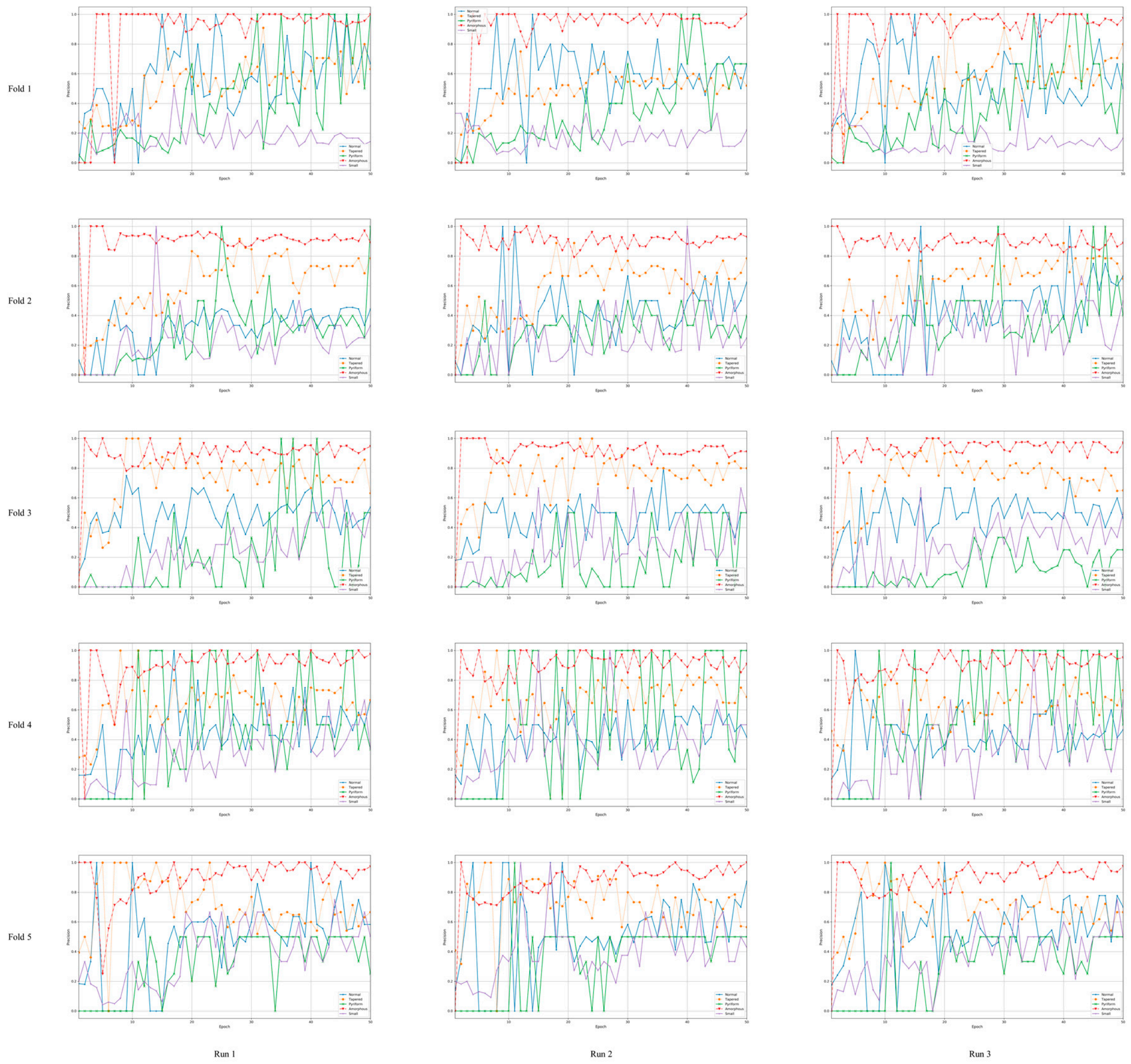

Figure S12. Detailed experimental results of the proposed model through 50 epochs in the total agreement setting of the SCIAN dataset, where 15 precision curves of each class with the number of epochs during the training on each of five possible choices of the test sets for 3 runs (5 folds × 3 runs) are illustrated.

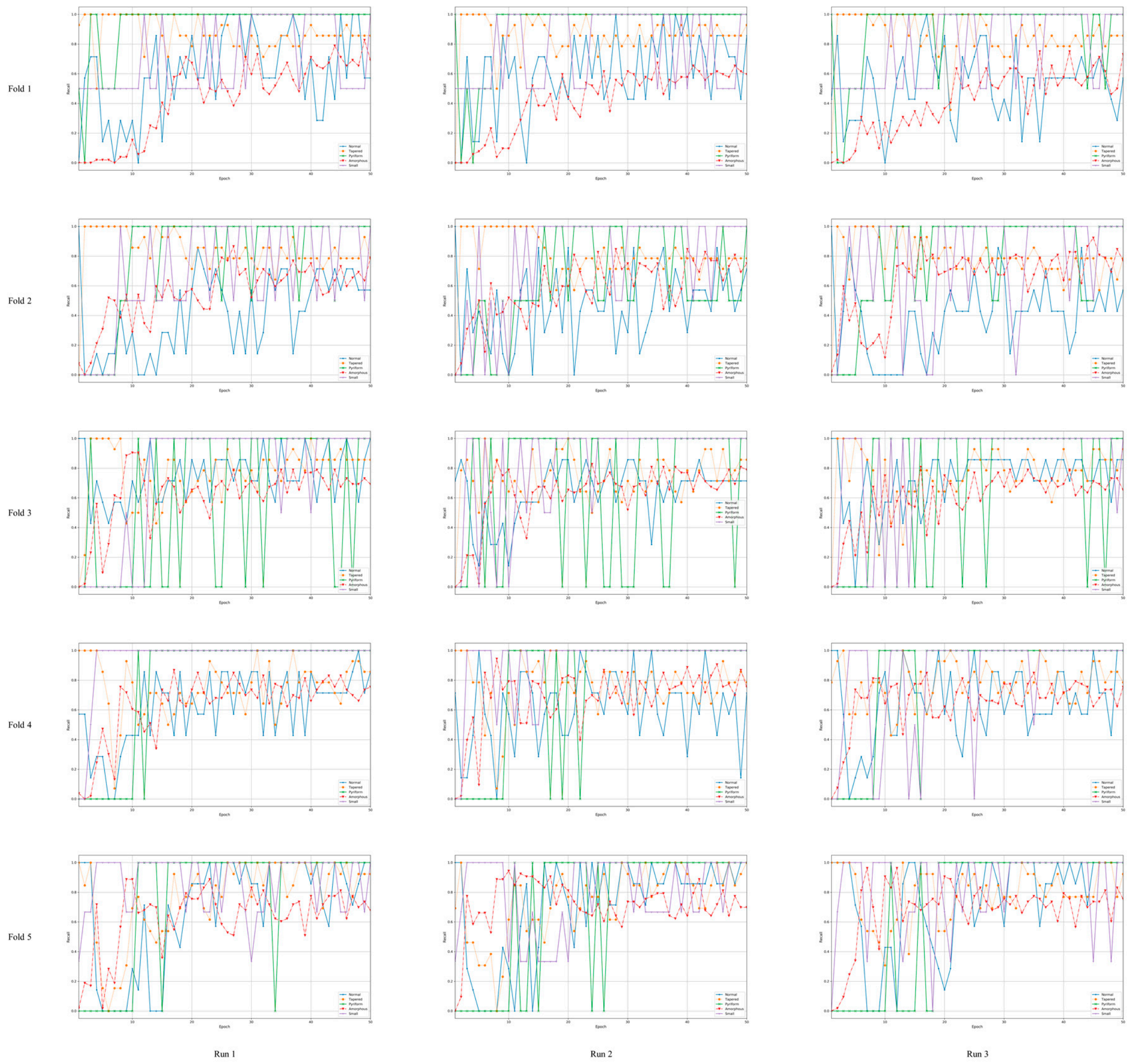

Figure S13. Detailed experimental results of the proposed model through 50 epochs in the total agreement setting of the SCIAN dataset, where 15 recall curves of each class with the number of epochs during the training on each of five possible choices of the test sets for 3 runs (5 folds × 3 runs) are illustrated.

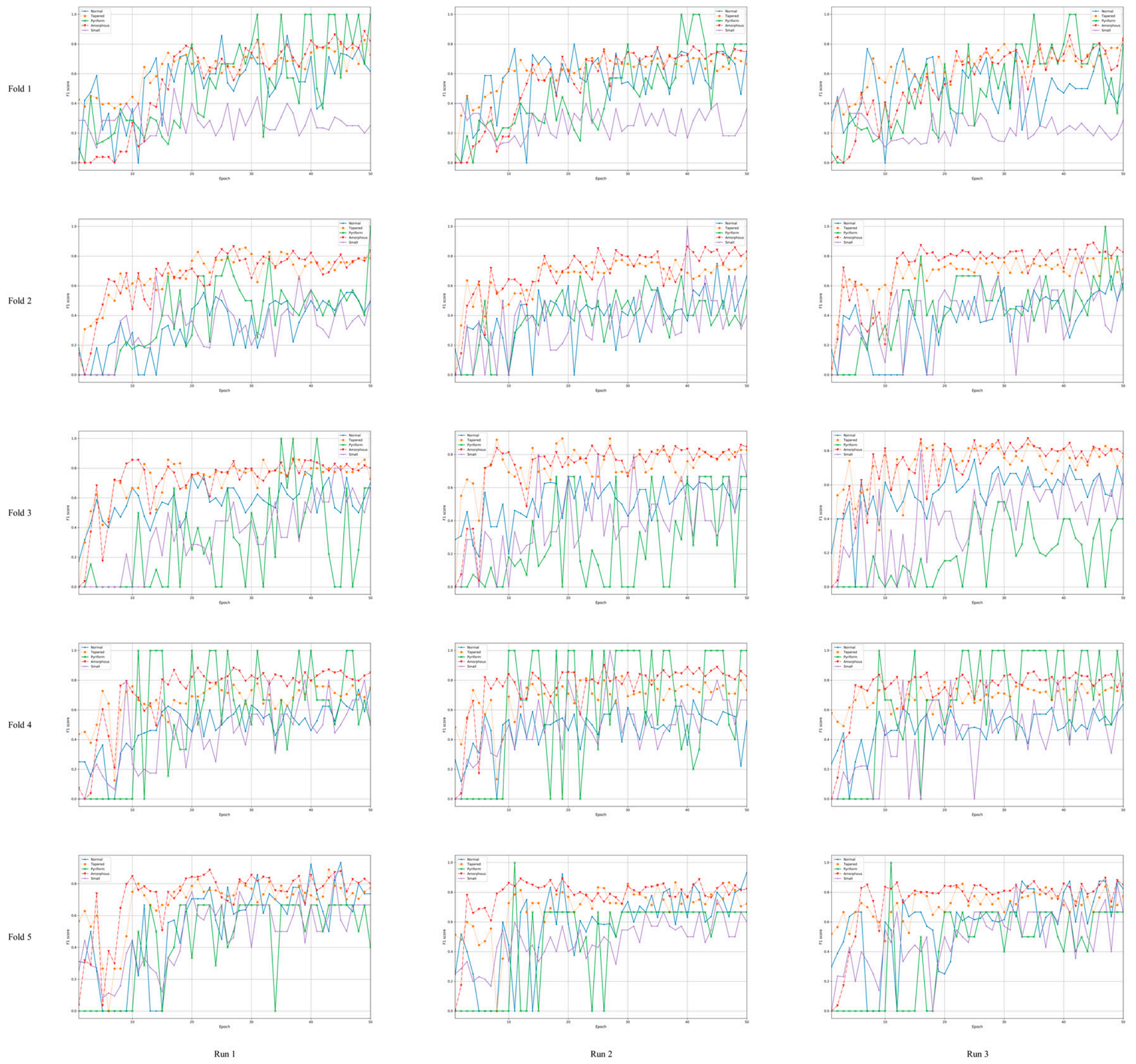

Figure S14. Detailed experimental results of the proposed model through 50 epochs in the total agreement setting of the SCIAN dataset, where 15 F_1-score curves of each class with the number of epochs during the training on each of five possible choices of the test sets for 3 runs (5 folds × 3 runs) are illustrated.

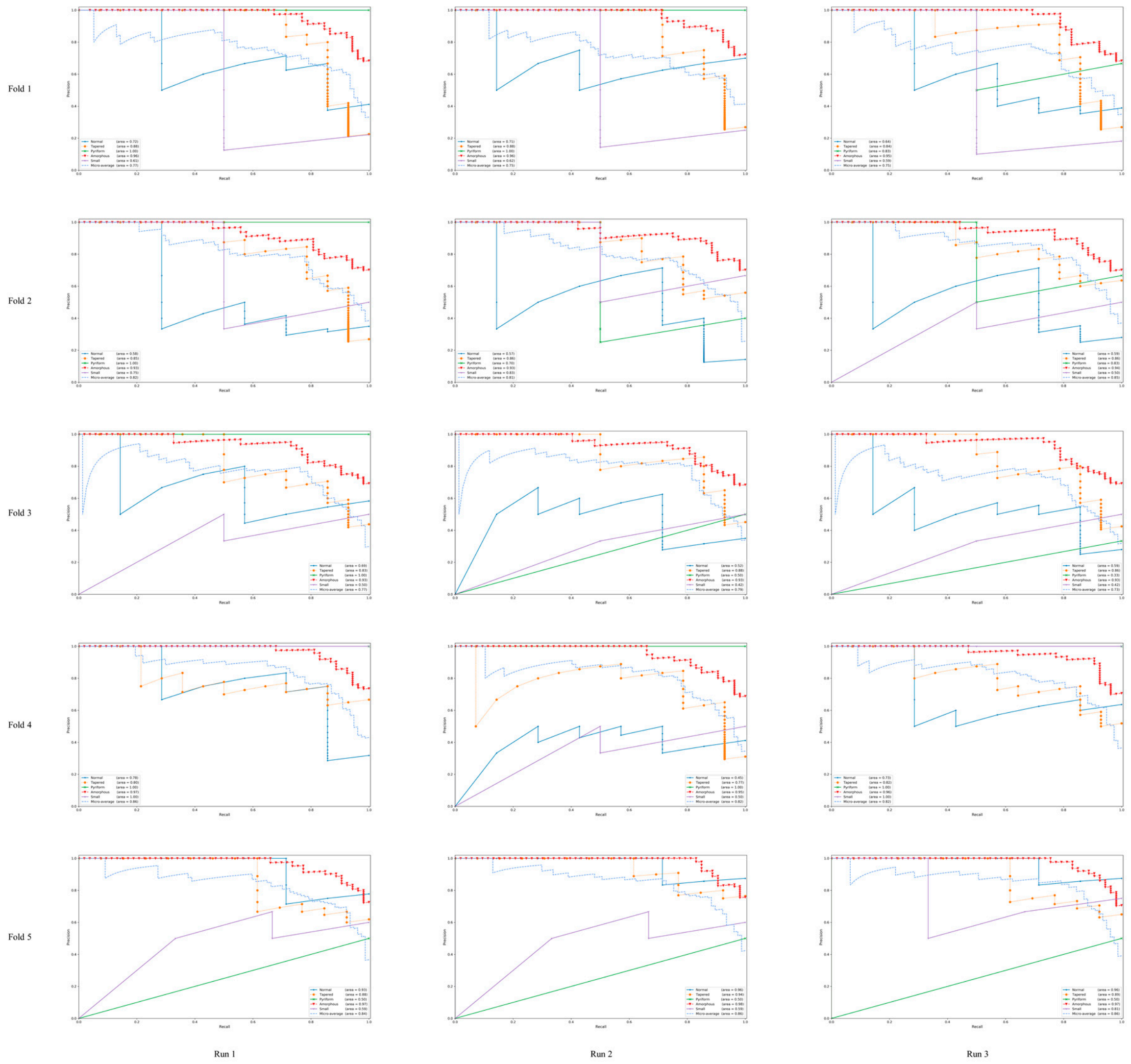

Figure S15. Detailed experimental results of proposed model in the total agreement setting of the SCIAN dataset, where 15 precision-recall curves of each class and their micro-averaging precision-recall curve on each of five possible choices of the test sets for 3 runs (5 folds $\times$ 3 runs) are illustrated. A high area under the curve signifies the high precision as well as high recall.

Fold 1
Fold 2
Fold 3
Fold 4
Fold 5

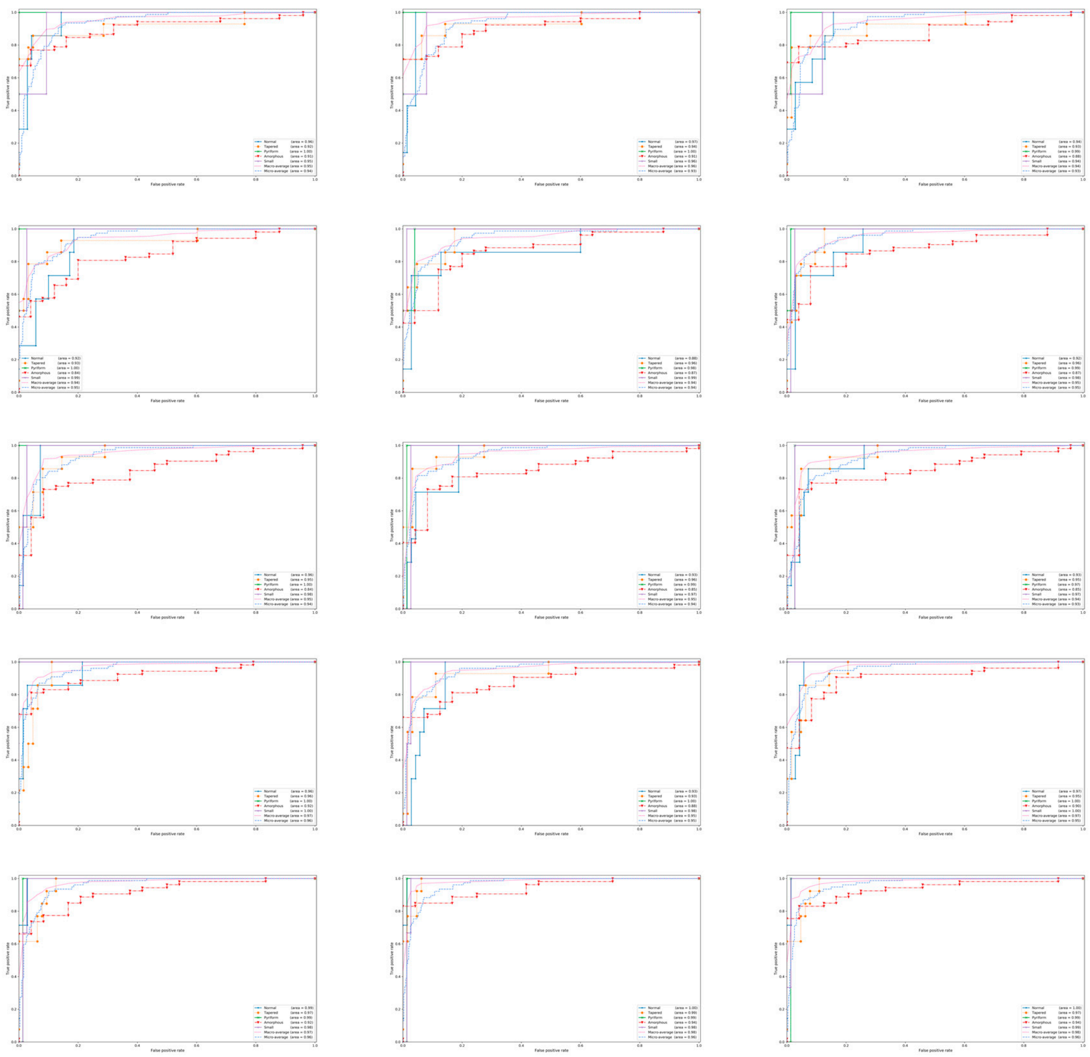

Run 1 Run 2 Run 3

Figure S16. Detailed experimental results of proposed model in the total agreement setting of the SCIAN dataset, where 15 receiver operating characteristic (ROC) curves of each class and their macro and micro-averaging ROC curves on each of five possible choices of the test sets for 3 runs (5 folds × 3 runs) are illustrated. These plots show the tradeoff between the true positive rate and false positive rate.

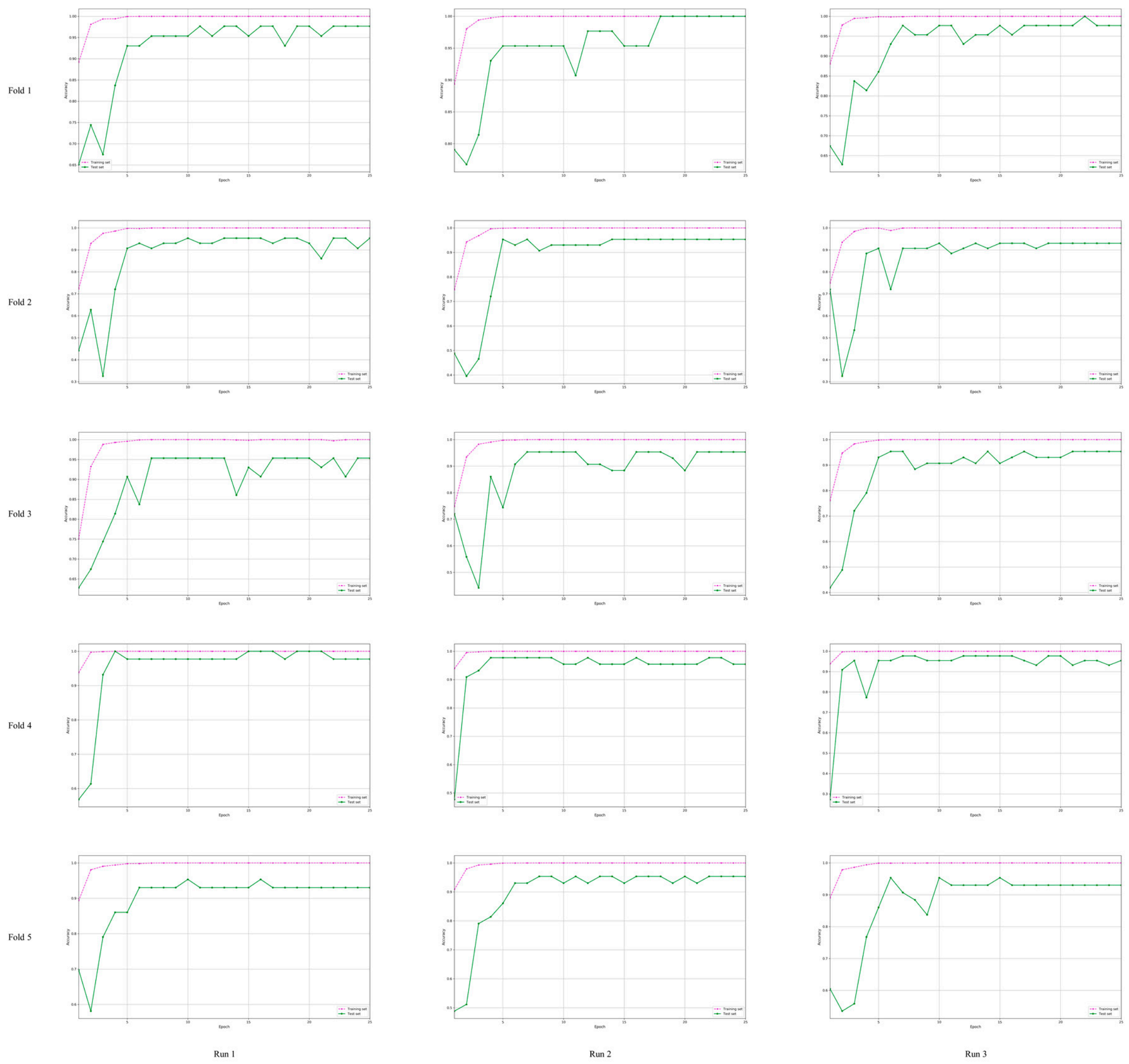

Figure S17. Detailed experimental results of the proposed model through 25 epochs of the HuSHeM dataset, where 15 classification accuracy curves with the number of epochs during the training on each of five possible choices of the training and test sets for 3 runs (5 folds $\times$ 3 runs) are illustrated.

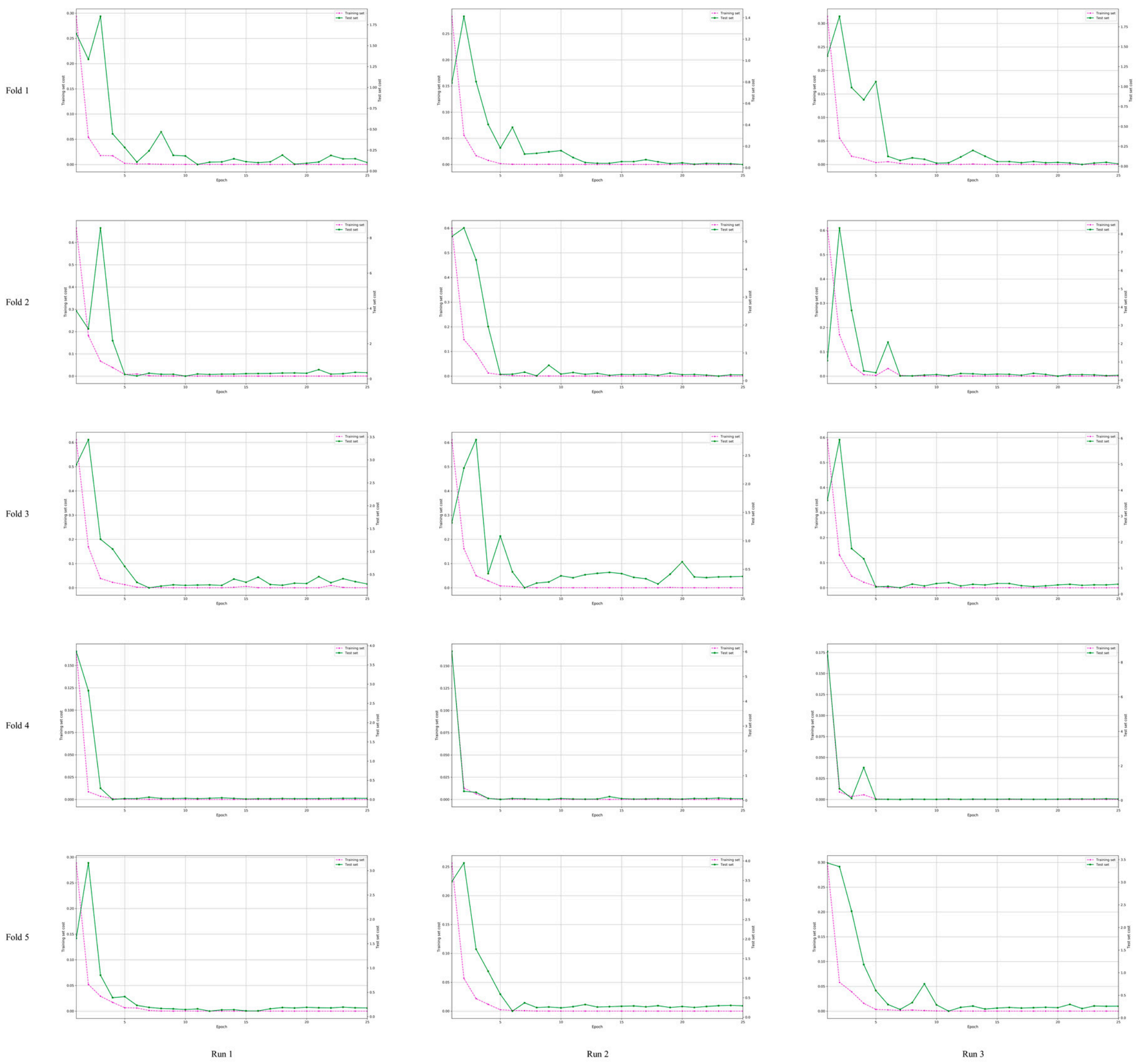

Figure S18. Detailed experimental results of the proposed model through 25 epochs of the HuSHeM dataset, where 15 cost curves with the number of epochs during the training on each of five possible choices of the training and test sets for 3 runs (5 folds $\times$ 3 runs) are illustrated.

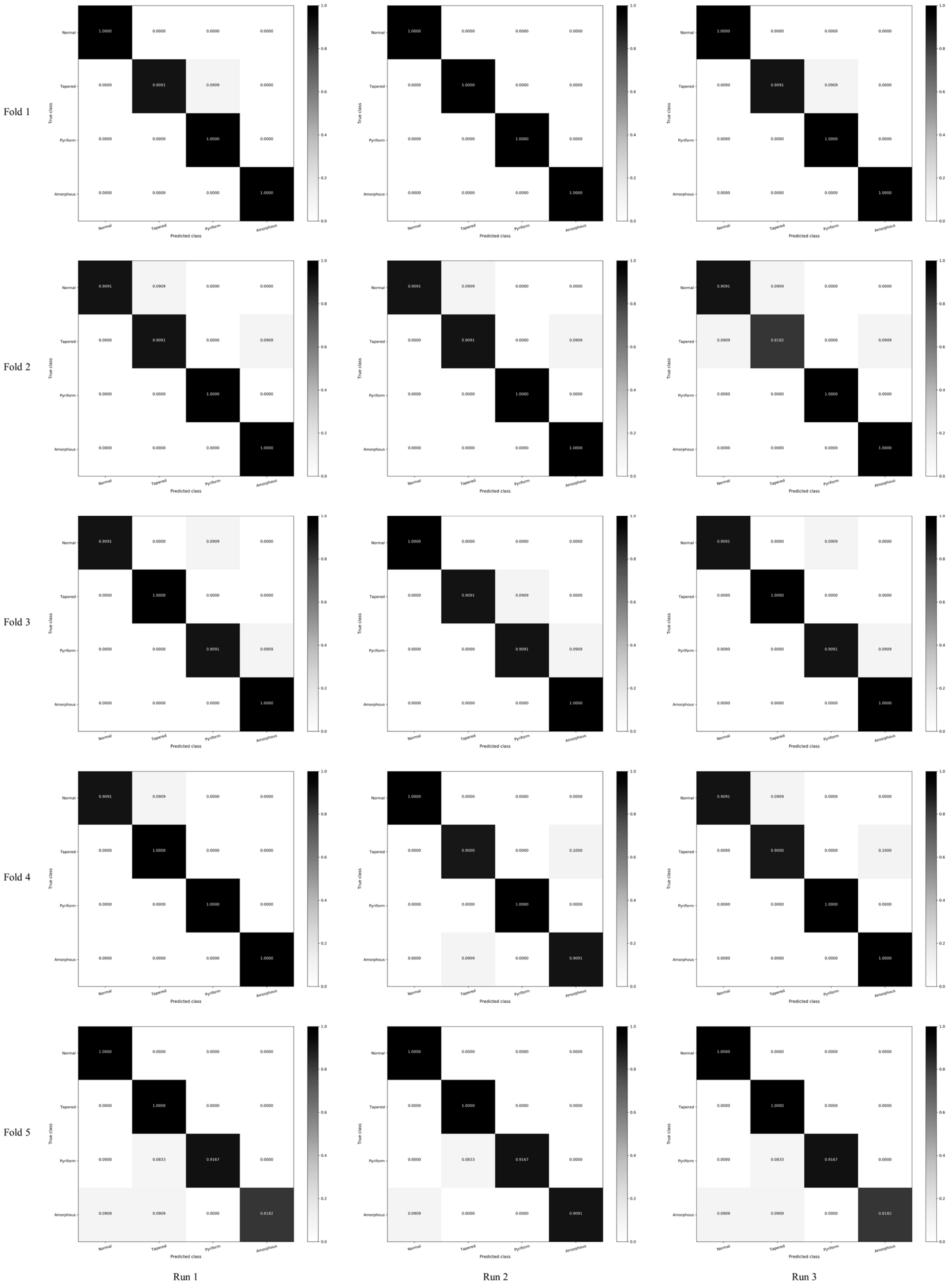

Figure S19. Detailed experimental results of the proposed model through 25 epochs of the HuSHeM dataset, where 15 confusion matrixes on each of five possible choices of the test sets for 3 runs (5 folds × 3 runs) are illustrated.

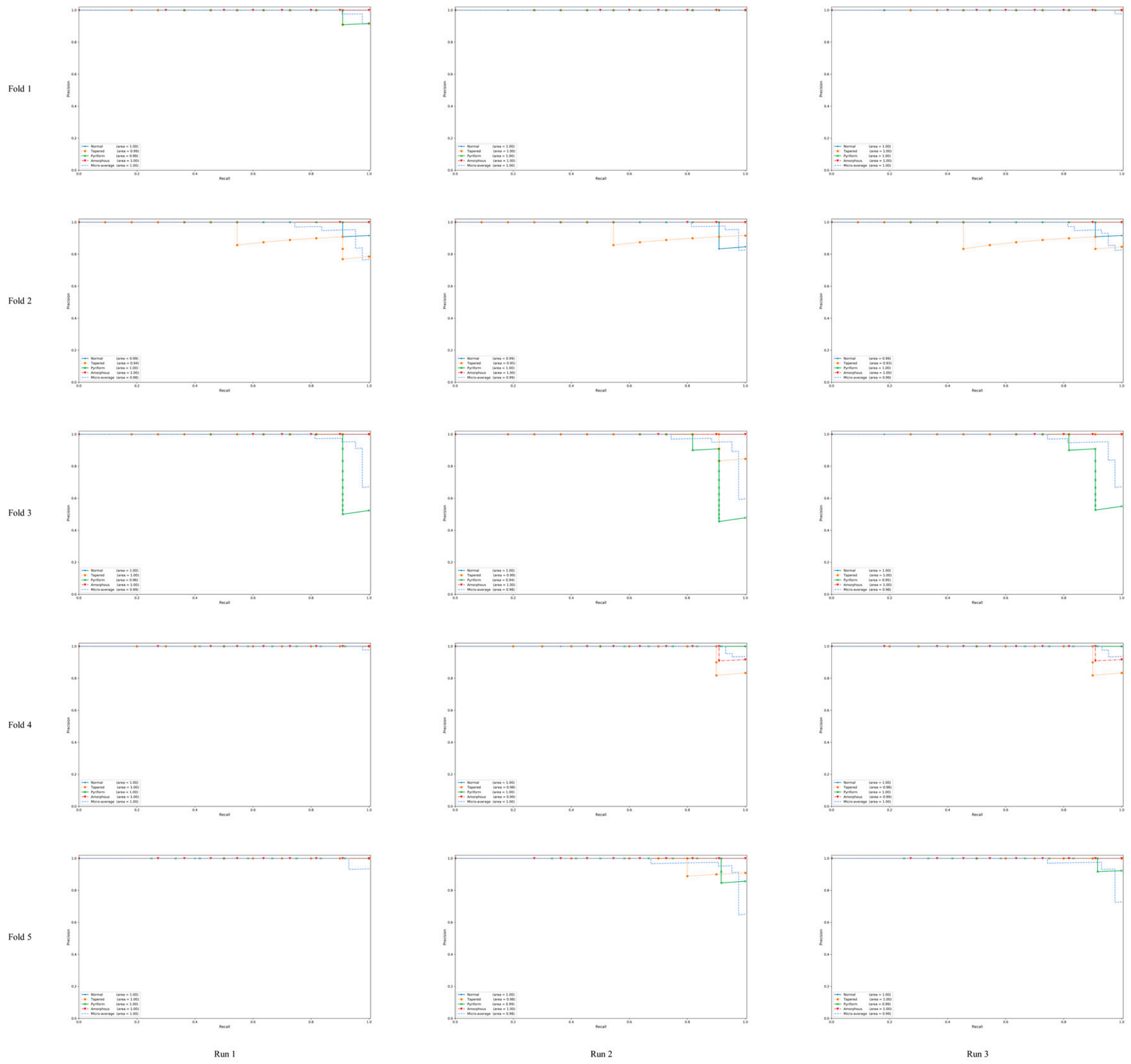

Figure S20. Detailed experimental results of proposed model of the HuSHeM dataset, where 15 precision-recall curves of each class and their micro-averaging precision-recall curve on each of five possible choices of the test sets for 3 runs (5 folds $\times$ 3 runs) are illustrated. A high area under the curve signifies the high precision as well as high recall.

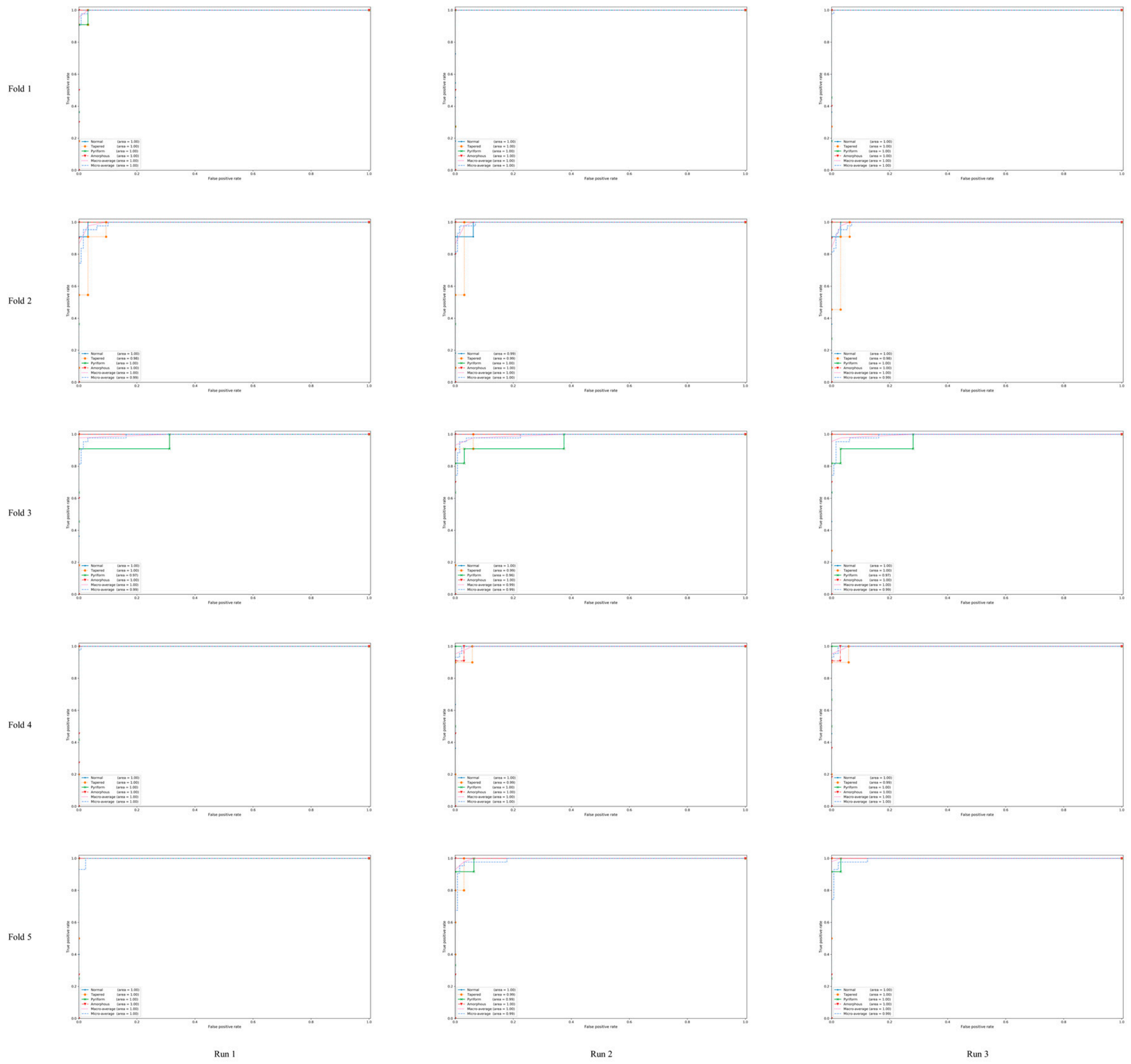

Figure S21. Detailed experimental results of proposed model of the HuSHeM dataset, where 15 receiver operating characteristic (ROC) curves of each class and their macro and micro-averaging ROC curves on each of five possible choices of the test sets for 3 runs (5 folds $\times$ 3 runs) are illustrated. These plots show the tradeoff between the true positive rate and false positive rate.
